# Selenite Interacting with Thiols Catalytically Releases NO from GSNO, Cleaves Plasmid DNA and Reduces the Hypotensive Effect of GSNO in Rats

**DOI:** 10.1007/s12011-026-05105-6

**Published:** 2026-04-14

**Authors:** Anton Misak, Marian Grman, Lenka Tomasova, Miroslav Chovanec, Karol Ondrias

**Affiliations:** 1 Institute of Clinical and Translational Research, Biomedical Research Center, Slovak Academy of Sciences, Dubravska cesta 9, Bratislava, 84505 Slovak Republic; 2 Cancer Research Institute, Biomedical Research Center, Slovak Academy of Sciences, Dubravska cesta 9, Bratislava, 84505 Slovak Republic

**Keywords:** S-nitrosoglutathione, Selenite, Selenomethionine, Nitric oxide, DNA cleavage, Rat blood pressure

## Abstract

Selenium (Se), an essential trace element, plays a significant role in human health. This study aimed to explain the biological effects of selenite (SeO_3_^2−^) and selenomethionine (SeMet), two Se forms commonly used as dietary supplements, in processes involving nitric oxide (NO) release. We found that products formed by the interaction of SeO_3_^2−^ or SeMet with excess glutathione (GSH), cysteine or homocysteine, catalytically release NO from S-nitrosoglutathione (GSNO) in vitro at physiologically relevant SeO_3_^2−^concentrations observed in blood. Administration of GSNO into the jugular vein of hypertensive rats decreased systolic and diastolic blood pressure (BP), heart rate, and augmentation index. However, these effects were markedly attenuated when GSNO was pre-incubation with the GSH/SeO_3_^2−^ mixture prior to administration. Furthermore, SeO_3_^2−^ combined with cysteine, homocysteine, GSH and N-acetylcysteine did not induce plasmid DNA (pDNA) cleavage in vitro. However, the presence of GSNO in the SeO_3_^2−^/thiol mixture caused pDNA cleavage in a bell-shaped manner depending on GSNO concentration. We suggest that under suitable biological conditions thiol/Se-derivatives release NO from NO-donors, but part of the released NO subsequently interacts with the interaction products, resulting in decreased NO bioavaibility in situ. These thiol/SeO_3_^2–^ interactions with NO-donors affect NO-dependent vascular activity and DNA stability. In conclusion, the observed effects may explain selenium’s dual roles in cardiovascular regulation and cancer prevention.

**Supplementary Information:**

The online version contains supplementary material available at 10.1007/s12011-026-05105-6.

## Introduction

Selenium (Se), an essential trace element, plays a crucial role in human health. Its levels in the organism strongly depend on dietary intake [1, 2]. In cases of Se deficiency in the diet, inorganic selenite (SeO_3_^2−^) and organic selenomethionine (SeMet) are mostly used as dietary supplements. Both beneficial and detrimental effects of Se consumption are well-known [3, 4]. The protective role of Se in several diseases has been documented [1, 2, 4–8]. However, chronic overconsumption of Se may lead to intoxication, compromised functions of several organs and has been associated with the development of cancer, cardiovascular and liver disease [1, 2, 4, 9–11]. Nitric oxide (NO) is involved in multiple physiological processes [12–22]. S-nitrosoglutathione (GSNO) is an endogenous donor of NO and a natural NO depot in biological systems [23]. It participates in S-nitrosylation, a post-translational modification that regulates protein function but may also cause protein misfolding, thereby contributing to oxidative stress, mitochondrial dysfunction and cell apoptosis [24–29]. Since endogenous and exogenous GSNO exert numerous positive or negative biological effects *via* NO release, compounds that promote NO release have been studied [30–36]. Selenocystamine, selenocystine, organoselenium-derivatized polymers and Se nanoparticles, can catalyze NO release from GSNO or from NO prodrugs in the presence of thiols [3, 37, 38].

Several reports indicate that SeO_3_^2–^ influences NO bioavailability and, consequently, NO-associated physiological effects. It has been reported that increased levels of NO products in diabetic rats decreased to control levels after treatment with SeO_3_^2–^ [39]. Subcutaneous injection of high concentration of SeO_3_^2–^ led to severe oxidative damage in the lenticular tissues in rats, as shown by elevated generation of NO radicals and elevated levels of the NO synthase gene and protein expression [40]. Intraperitoneal injection of SeO_3_^2–^ tended to increase plasmatic NO concentration in control rats, but in lipopolysaccharide-treated rats, it decreased plasmatic NO [41]. SeO_3_^2–^ also increased serum NO levels in atherosclerotic model rats [42]. Incubation of endothelial cells with high SeO_3_^2–^ concentrations (5 and 10 µmol/L) reduced the production of NO, measured as reduction in nitrite concentrations in culture media, compared to control and low SeO_3_^2–^ concentration (0.5 µmol/L) conditions [43]. In vitro, at concentrations higher than 2–5 µmol/L, SeO_3_^2–^ reduced the production of NO in activated cells by inhibiting NF-κB binding to DNA through an oxidative mechanism [44]. Similarly, mRNA expression and activity of inducible NO synthase, as well as NO content, were significantly increased in diabetic mice, whereas these parameters decreased in SeO_3_^2–^-treated diabetic mice [45]. SeO_3_^2–^ (0.625 mg/kg) injected intraperitoneally 2 h before induction of ischemia in rats did not significantly change NO tissue level [46]. In addition, SeO_3_^2–^ modified NO levels in plants [47]. NO has been shown to play a dual role in regulating DNA damage response signaling in pancreatic β-cells [48]. Taken together, these diverse findings showing both increased and decreased NO bioavailability induced by SeO_3_^2–^ indicate that its interaction with NO donors is not yet fully understood.

Similarly, the relationship between selenium concentration and blood pressure remains controversial. Some studies have indicated a ‘threshold effect’, U-shaped relationship, or no correlation between the Se levels and blood pressure (BP), while the others have reported either negative or positive relationship, leading to an increased incidence of hypertension [49–54].

Therefore, the aim of the present study was to clarify the numerous and sometimes contradictory biological effects of SeO_3_^2–^ and SeMet in processes involving NO. We have investigated in detail the catalytic potency of thiol/selenite and thiol/SeMet products to release NO from GSNO in vitro, determining which concentrations of SeO_3_^2–^ or SeMet are capable of releasing NO from GSNO in the presence of thiols. We examined whether these concentrations are physiologically relevant and how products of the glutathione (GSH)/SeO_3_^2–^/GSNO reaction, after releasing NO, influence rat cardiovascular function in vivo and plasmid DNA cleavage in vitro.

## Materials and Methods

### Chemicals and Solutions

The following chemicals were purchased from Sigma-Aldrich (Schnelldorf, Germany): sodium selenite (SeO_3_^2–^; 214485), L-cysteine hydrochloride (Cys; C1276), DL-homocysteine (HCys; H4628), L-cystine dihydrochloride (cystine; C6727), L-glutathione reduced (GSH; G4251), glutathione oxidized (GSSG; G6654), N-acetyl-L-cysteine (NAC; A7250), L-methionine (MET; M9625), Griess reagent (G4410), S-Nitrosoglutathione (GSNO; N4148), sodium nitrite (NaNO_2_^–^, 431605), diethylenetriaminepentaacetic acid (DTPA, D6518), superoxide dismutase (SOD; S7446-15KU), catalase (CAT; C9322), 2-(4-carboxyphenyl)-4,5-dihydro-4,4,5,5-tetramethyl-1*H*-imidazol-1-yloxy-3-oxide potassium salt (cPTIO; C221), sodium phosphate monobasic (NaH_2_PO_4_, S5011) and sodium phosphate dibasic (Na_2_HPO_4_, S7907). Seleno-L-methionine (SeMet; 1611955) was from United States Pharmacopeia. Isoflurin (Isoflurane, 1,000 mg/g) was purchased from Vetpharma (Barcelona, Spain). pBR322 plasmid (New England BioLabs, Inc., N3033 L, Ipswich, MA, USA).

### Measurement of Thiols/SeO_3_^2–^ Potency to Release NO from S-Nitroglutathione

Stock solutions of the studied compounds were prepared fresh every day and added in volume (1–50 µL) to the appropriate volume (999 − 950 µL) of 100 mmol/L sodium phosphate, 100 µmol/L DTPA buffer, pH 7.4, 37 °C containing the final concentration of 100 µmol/L GSNO. Cystine (10 mmol/L) was prepared 5 s before application. UV-Vis absorption spectra (900–220 nm) were recorded every 30 s (or 1 min) for 30 min (or 120 min) with a Shimadzu UV-1800 spectrometer (Kyoto, Japan) at 37 °C. In all experiments, the cuvettes with optical path length of 10 mm were used. The kinetics of GSNO decomposition was measured as the decrease in absorbance (ABS) at 334 nm [55]. ABS values at 334 nm were obtained after subtracting ABS at 500 nm. To compare the rates of NO release, time-resolved decreases of ABS at 334 nm were fitted using the exponential decay equation: *ABS(t)* = *ABS*_0_ + *a* × *e*^− *b*×*t*^, where ABS_0_ represents ABS at initial time, *t* is the time in minutes. The parameters ‘*a*’ (arbitrary units) and ‘*b*’ (min^–1^) were evaluated from the fitted data, a higher ‘*b*’ means a higher rate of NO release from GSNO.

### Griess Assay

The Griess assay was used to measure the NO oxidation product, nitrite (NO_2_^–^) formed during reactions of SeO_3_^2–^, with GSH and Cys and during thiol/ SeO_3_^2–^ interactions with GSNO [31, 55]. SeO_3_^2–^ (10 µmol/L, final) was added to GSNO solution (100 µmol/L, final) in phosphate buffer, followed by Cys or GSH addition (total volume of 125 µL). Samples were incubated at 32 ± 1 °C for 15 min. Then 125 µL of the Griess reagent (stock, 400 mg in 10 ml distilled H_2_O) was added to quantify NO_2_^–^. After 10 min incubation absorbance spectra in the wavelength range of 450–700 nm (5 nm step) were recorded by a Synergy H1 Spectrophotometer (BioTek Instruments, Inc., USA) in polystyrene microplates. Data were acquired using Gen5 Data Analysis software (BioTek Instruments, Inc., USA). ABS at 548 nm was used for quantification of NO release (NO_2_^−^ formation), based on calibration curve generated with known concentrations of NaNO_2_.

### Rats In Vivo Experiments

Adult male spontaneously hypertensive rats (SHRs) (*n* = 6; 280–320 g; 17–19 weeks old) were purchased from the Department of Toxicology and Laboratory Animals Breeding, Centre of Experimental Medicine, Dobra Voda and housed at the Central Animal House Facility of Pavilion of Medical Sciences (registration number SKUCH 04022, Bratislava, Slovak Republic). Animals received veterinary care as described by Balis and co-workers [56]. Isoflurane (ISO) inhalational anesthesia, surgical procedures, recording of arterial pulse waveform (APW) and evaluation of APW parameters (APW-Ps) were performed as described in our previous study [57]. Briefly, the right jugular vein of anesthetized SHR rats was cannulated for administration of compounds and the left carotid artery was cannulated for the detection of APW from which six APW parameters were evaluated using a microcatheter pressure transducer [58].

Stock solutions of thiols, prepared in 100 mmol/L phosphate buffer, 100 µmol/L DTPA, 7.4 pH and SeO_3_^2–^ in 0.9% NaCl were used within day. GSNO was prepared in distilled H_2_O, stored at − 80 °C and freshly dissolved prior to administration. To prepare the GSH/SeO_3_^2−^/GSNO mixture, 100 µL of 40 mmol/L GSH, 40 µL of 10 mmol/L SeO_3_^2–^ and 4 µl of 8 mmol/L GSNO were added to 56 µL phosphate puffer, mixed and incubated for 20 s at 23 ± 1 °C. The resulting stock concentrations of the GSH/SeO_3_^2−^/GSNO mixture were 20/2/0.16 in mmol/L. After the incubation, the mixture was administered intravenously (IV) into the cannulated right jugular vein at a bolus dose of 500 µL/kg over 15 s. The calculated dose of the GSH/SeO_3_^2−^/GSNO mixture was 10/1/0.08 µmol/kg in rat. The analog signal was analyzed to identify ten APW points (a-j) indicated in Supplementary Information Fig. S1. Definition and abbreviation of eight APW-Ps calculated from the APW points are provided in Supplementary Information Fig. S1.

### Plasmid DNA Cleavage

A plasmid DNA (pDNA) cleavage assay using pBR322 plasmid was performed as reported previously [59]. In this assay, each sample contained 0.2 µg pDNA in 20 µL sodium phosphate buffer (25 mmol/L sodium phosphate, 50 µmol/L DTPA, pH 7.4). To 10 µL of the pDNA solution (0.2 µg in 50 mmol/L sodium phosphate containing 100 µmol/L DTPA), 2.5 µL of a SeO_3_^2−^ stock solution (240 µmol/L in H_2_O) was added, followed by 5 µL of GSNO (0-960 µmol/L in H_2_O), and finally 2.5 µL of the thiol stock solution (Cys, GSH, HCys, and NAC at 960 µmol/L in H_2_O). Mixtures were incubated at 37 °C for 30 min and then subjected to 0.6% agarose gel electrophoresis. Integrated densities of pBR322 forms were quantified using Image Studio analysis software (LI-COR Biotechnology, Bad Homburg, Germany) to determine pDNA cleavage efficiency.

## Results

### The Mixture of SeO_3_^2–^ with Cys or HCys Catalyzes Release of NO from GSNO

**Fig. 1:**
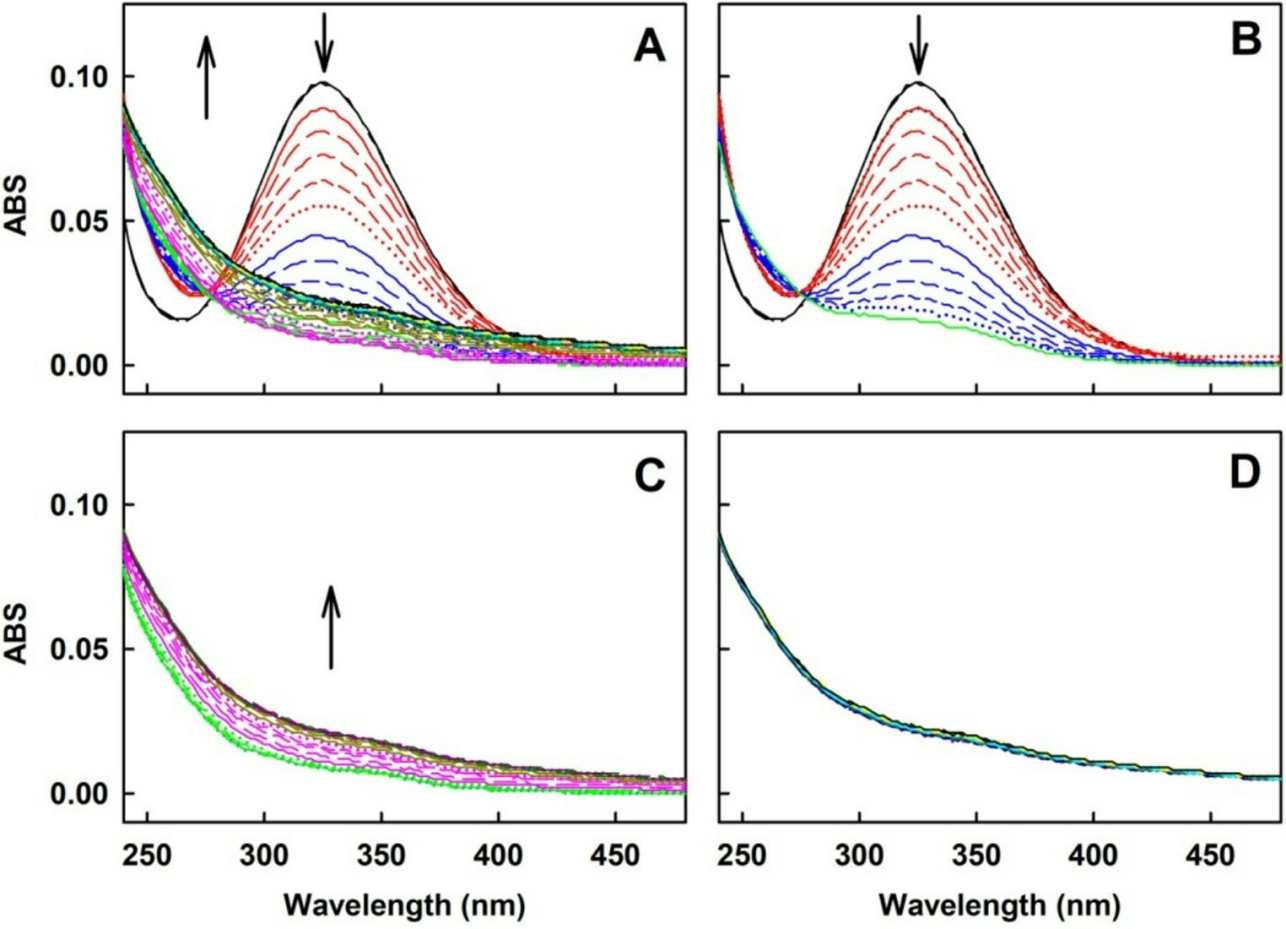
Representative time resolved UV-Vis spectra of the interaction of SeO_3_^2–^ with GSNO in the presence of Cys. The decrease in ABS at 334 nm indicates the release of NO from GSNO. UV-Vis spectra of GSNO (100 µmol/L, final) was measured every 30 s for 1.5 min in 100 mmol/L sodium phosphate, 100 µmol/L DTPA, pH 7.4, 37 °C (black lines) followed by subsequent addition of SeO_3_^2–^ (10 µmol/L, final) and Cys (200 µmol/L, final) 15 s later and measured every 30 s for 30 min. The solid red line indicates the first spectrum after addition of SeO_3_^2–^/Cys, which is followed each 30 s by: long dash red, medium dash red, short dash red, dotted red, solid blue line, long dash blue, medium dash blue, etc. Time resolved UV-Vis spectra for 0–30 min (**A**). Time resolved UV-Vis spectra for 0–5.5 min (**B**), for 6–12 min (**C**) and for 12.5–30 min (**D**)

In control experiments, UV-Vis spectra and ABS at 334 nm of 100 µmol/L GSNO either alone or in the presence of 100 µmol/L SeO_3_^2–^ remained stable in the buffer solution for 30 min, indicating that GSNO is relatively stable and did not react with SeO_3_^2–^ (Fig. S2). Cys (200 or 1 000 µmol/L) or GSH (10 mmol/L) for 30 min had only minor effect to release NO from GSNO (Fig. S3). Addition of 10 µmol/L SeO_3_^2–^ into the GSNO/Cys mixture (100/200 µmol/L) induced time-dependent spectral changes (Fig. 1A). ABS at 334 nm decreased within ~ 0–5.5 min indicating total GSNO decomposition (Fig. 1B). This was followed by a gradual increase in ABS between ~ 6–12 min in the ~ 250–600 nm range (Fig. 1C) indicating the occurrence of complex chemical reaction(s) among the components of the mixture. At later time points (~ 13–30 min), no further changes in ABS of the spectra were observed, suggesting the absence of additional time dependent chemical reactions (Fig. 1D). Examples of the complex time-dependent reactions of GSNO/Cys/SeO_3_^2–^ (100/200/30 in µmol/L) mixture are shown in Fig. S4 and reactions of the Cys/SeO_3_^2–^ (200/100 in µmol/L) or GSNO/Cys/SeO_3_^2–^ (100/200/100 in µmol/L) mixtures are presented in Fig. S5.

**Fig. 2:**
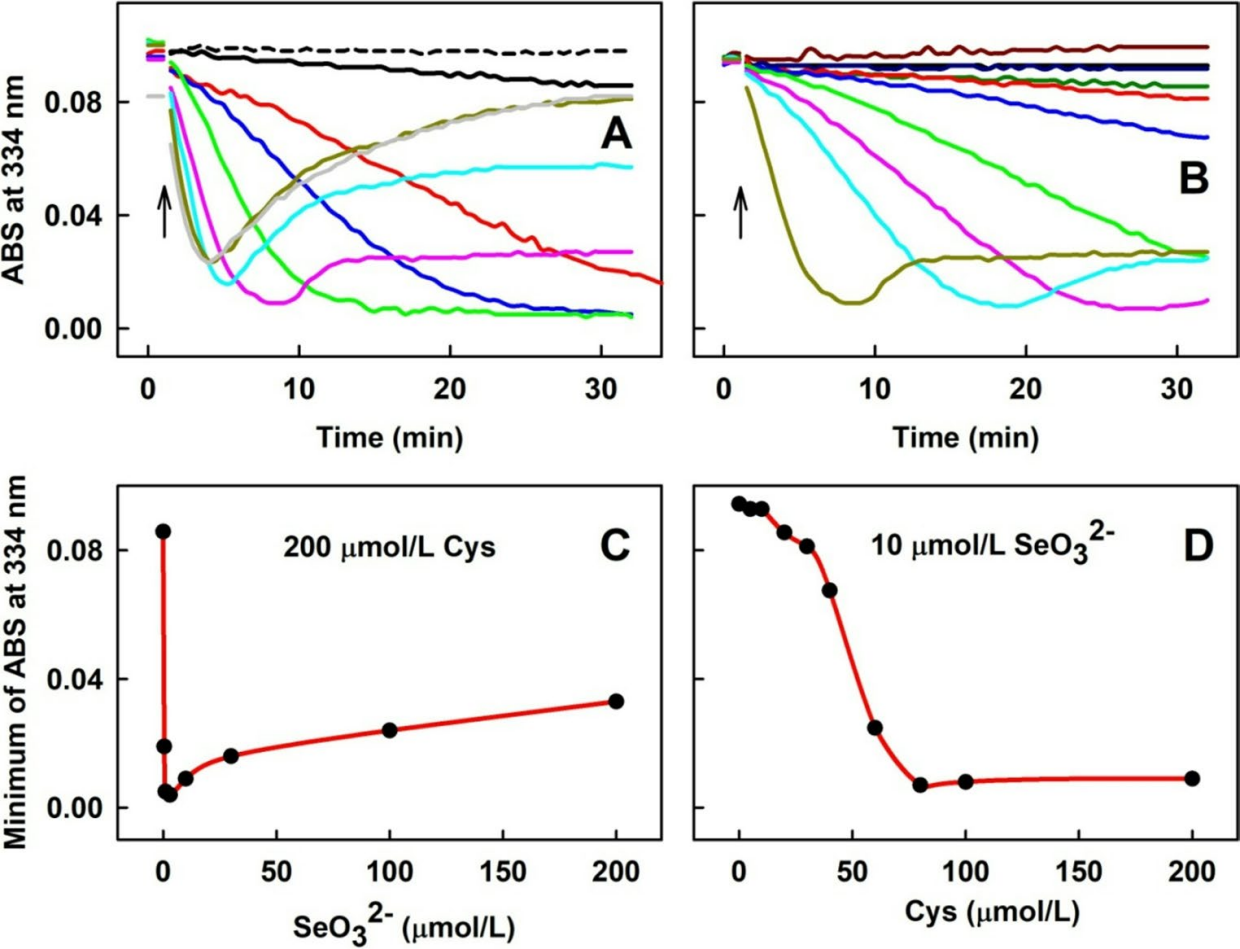
Time-dependent ABS at 334 nm of UV-Vis spectra during the interaction of SeO_3_^2–^/GSNO/Cys. The decrease in ABS at 334 nm indicates the release of NO from GSNO. (**A**) Time-dependent ABS of 100 µmol/L GSNO (dash black) and effects of GSNO/Cys (100/200 in µmol/L) without (black) and with increased SeO_3_^2–^ concentrations; 0.5 (red), 1 (blue), 3 (green), 10 (pink), 30 (cyan), 100 (dark yellow) and 200 µmol/L (gray). Decrease of ABS at 334 nm represents NO release from GSNO (see also Fig. 1B). Later, ABS increased, showing chemical interaction of the mixture components (see also Fig. 1C). (**B**) Time-dependent effect of SeO_3_^2–^/GSNO (10/100 in µmol/L) without (black) and with increased Cys concentrations; 5 (dark red), 10 (dark blue), 20 (dark green), 30 (red), 40 (blue), 60 (green), 80 (pink), 100 (cyan) and 200 µmol/L (dark yellow). (**C**) Concentration-dependent effect of SeO_3_^2–^ on NO release from GSNO (minimum of ABS at 334 nm during 30 min of measurement) in the mixture of GSNO/Cys (100/200 in µmol/L), data are derived from panel (A). (**D**) Concentration-dependent effect of Cys on NO release from GSNO (minimum of ABS at 334 nm during 30 min of measurement) in the mixture of SeO_3_^2–^/GSNO (10/100 in µmol/L), data are derived from panel (**B**)

As indicated by the decrease of ABS at 334 nm, the presence of SeO_3_^2–^ (0.5–100 µmol/L) in a GSNO/Cys (100/200 in µmol/L) solution induced NO release from GSNO (Figs. 2 and S6). The extent of NO release increased in a concentration-dependent manner within the 0.5-3 µmol/L SeO_3_^2–^ range. At higher SeO_3_^2–^ concentrations (10–100 µmol/L), the rate of the NO release was further accelerated (Fig. 2A, C). However, the extent of NO release could not be accurately evaluated at these concentrations, because after NO liberation, ABS at 334 nm increased again due to subsequent chemical reaction(s) among the mixture components, similarly to the pattern observed for the GSNO/Cys/SeO_3_^2–^ mixture in Fig. 1C. Cys also enhanced NO release in a concentration-dependent manner at constant concentrations of GSNO (100 µmol/L) and SeO_3_^2–^ (10 µmol/L). Little or only slow NO release was observed at Cys/SeO_3_^2–^ ratios ranging from 5/10 to 40/10 µmol/L. However, NO release significantly increased in a concentration-dependent manner when higher Cys/SeO_3_^2–^ ratios were used (Fig. 2B, D).

**Fig. 3:**
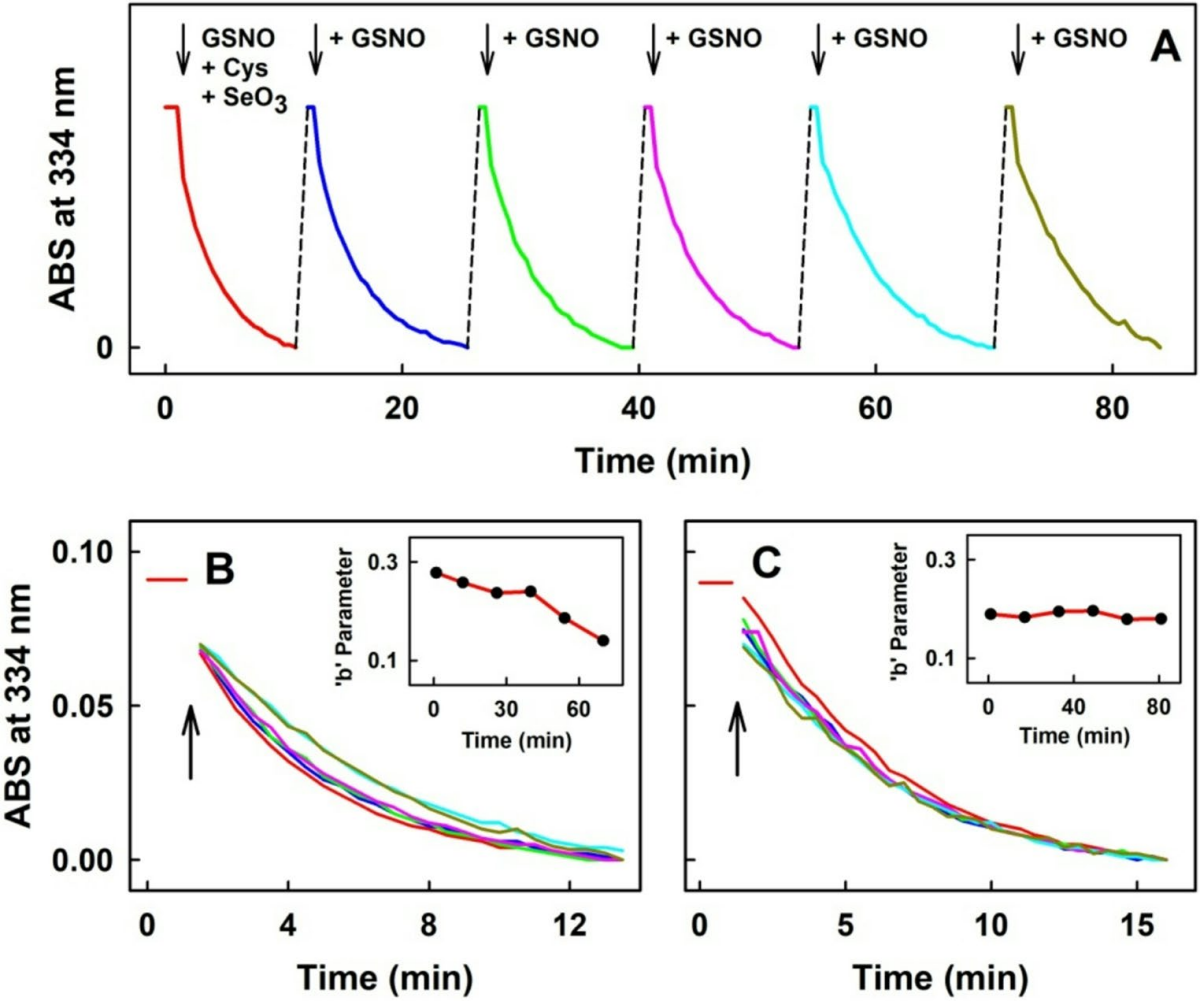
Time-dependent NO release from GSNO measured as ABS at 334 nm of UV-Vis spectra during the interaction of SeO_3_^2–^ with Cys. The decrease in ABS at 334 nm indicates the release of NO from GSNO. (**A**) Time-dependent effect of 100 µmol/L GSNO with the mixture of Cys/SeO_3_^2–^ (1 000/3 in µmol/L, red) and after adding of the 100 µmol/L GSNO five times in a row (blue, green, pink, cyan and dark yellow). (**B**) Details of the rate of decrease of ABS at 334 nm after six consecutive additions of GSNO to the Cys/SeO_3_^2–^ (1 000/3 in µmol/L) mixture. Data are derived from panel (**A**). (**C**) Details of the rate of decrease of ABS at 334 nm after six consecutive additions of GSNO to the HCys/SeO_3_^2–^ (1 000/3 in µmol/L) mixture. Additions of Cys and GSNO are marked by arrows

It was observed that the addition of 0.5 or 1 µmol/L SeO_3_^2–^ to the GSNO/Cys mixture resulted in complete NO release from 100 µmol/L GSNO (Fig. 2A). This findings suggests that product(s) of the SeO_3_^2–^/Cys interaction possesses catalytic property. Therefore, we studied whether the Cys/SeO_3_^2–^ mixture that had already released NO from GSNO was capable of inducing NO release from subsequently added GSNO. After the Cys/SeO_3_^2–^ (1 000/3 in µmol/L) mixture released NO from GSNO, an additional 100 µmol/L GSNO was introduced into the solution, resulting in further NO release. Similarly, complete NO release was observed after six consecutive additions of 100 µmol/L GSNO (Fig. 3A). The details of the NO release are shown in Fig. 3B. To compare the rate of the NO release after the GSNO additions, the parameter ‘b’ derived from fitted equation of exponential decay was used (Fig. 3B, Insert). The NO release rate was similar for the first three additions of GSNO, but decreased during the subsequent three additions (Fig. 3B, Insert). When a lower Cys/SeO_3_^2–^ ratio (200/3 in µmol/L) was used, the rate of NO release was already slower after the second GSNO addition and markedly reduced after the third addition (Fig. S7). Comparable catalytic behaviour, but with a slower rate of NO release from GSNO was observed when 100 µmol/L GSNO was added six consecutive times to the HCys/SeO_3_^2–^ (1 000/3 in µmol/L) mixture (Fig. 3C) compared with the Cys/SeO_3_^2–^ (1 000/3 in µmol/L) mixture (Fig. 3B).

### The Mixture of SeO_3_^2–^ with GSH Catalyzes Release of NO from GSNO

**Fig. 4:**
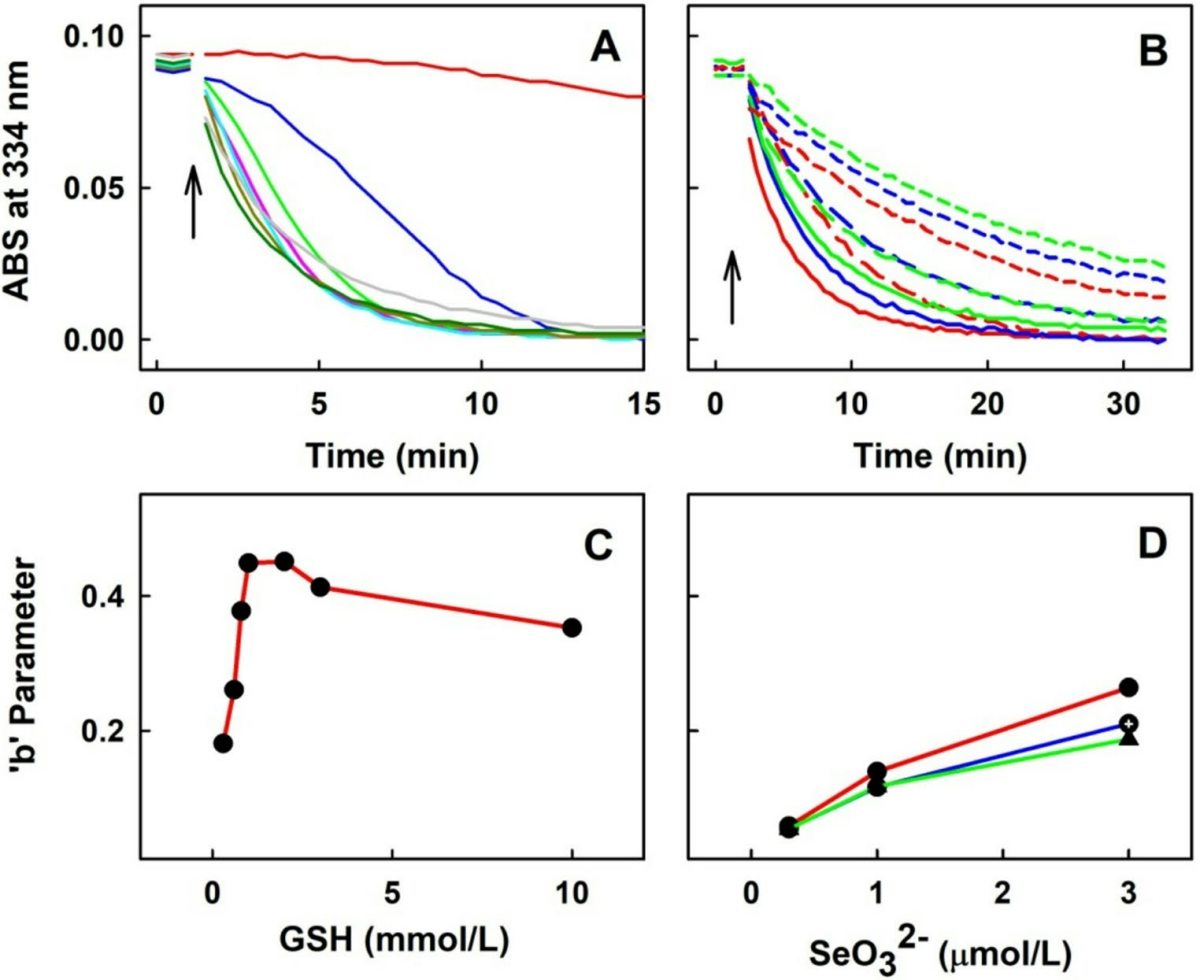
Time-dependent ABS at 334 nm of UV-Vis spectra during the interaction of GSH/SeO_3_^2–^/GSNO. The decrease in ABS at 334 nm indicates the release of NO from GSNO. (**A**) Time-dependence of SeO_3_^2–^/GSNO (10/100 in µmol/L) in the presence of increasing GSH concentrations: 100 (red), 300 (blue), 600 (green), 800 (pink), 1000 (cyan), 2000 (dark yellow) and 3000 µmol/L (gray). (**B**) Time-dependence of GSNO (100 µmol/L) in the presecnce of increasing SeO_3_^2–^ concentrations 0.3 (short dash lines), 1 (dash lines) and 3 µmol/L (solid lines) in the presence of 2 (red), 5 (blue) and 10 (green) mmol/L GSH. (**C**) Concentration-dependent effect of GSH on NO release from GSNO expressed as ‘b’ parameter, data are derived from panel (**A**). (**D**) Concentration-dependent effect of SeO_3_^2–^ on NO release from GSNO in the mixture of GSNO/GSH (100/2 000 in µmol/L, red), (100/5 000 in µmol/L, blue) and (100/10 000 in µmol/L, green), data are derived from panel (**B**)

We next investigated whether a thiol structurally different from Cys would have similar properties in terms of NO release from GSNO. Therefore, experimental analogous to those performed with Cys/SeO_3_^2–^ and HCys/SeO_3_^2–^ were performed using the GSH/SeO_3_^2–^ mixture. In control experiment, UV Vis spectra and ABS at 334 nm of 100 µmol/L GSNO with 10 mmol/L GSH showed that the GSH induced NO release from GSNO was minor (Fig. S3C). However, similarly to Cys, the presence of 10 µmol/L SeO_3_^2–^ in the GSH/SeO_3_^2–^ mixture released NO from GSNO (Fig. 4). At the constant concentration of SeO_3_^2–^ (10 µmol/L), the rate of the NO release increased with increasing concentrations of GSH (0.1-1 mmol/L), but decreased at higher concentrations (2–10 mmol/L) (Fig. 4A, C). At high GSH/SeO_3_^2–^ ratios, NO release increased with increasing SeO_3_^2–^ concentrations (0.3-3 µmol/L), but it slightly decreased with increasing concentration of GSH (2–10 mmol/L) (Fig. 4B, D).

**Fig. 5:**
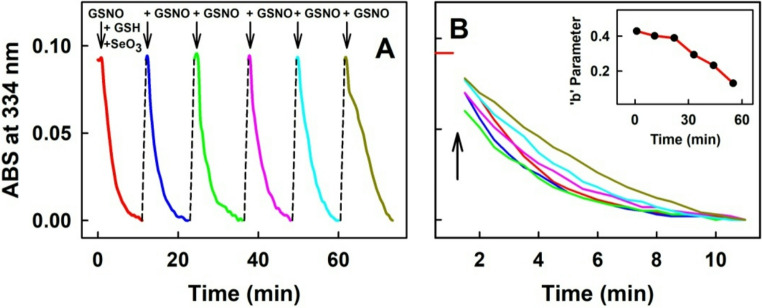
Time-dependent NO release from GSNO measured as ABS at 334 nm of UV-Vis spectra during the interaction of SeO_3_^2–^ with GSH. The decrease in ABS at 334 nm indicates the release of NO from GSNO. (**A**) Time-dependence of 100 µmol/L GSNO in the presence of a GSH/SeO_3_^2–^ (1 000/10 in µmol/L, red), followed by five successive additions of 100 µmol/L GSNO (blue, green, pink, cyan and dark yellow). (**B**) Details of the rate of decrease of ABS at 334 nm after six successive additions of GSNO into the GSH/SeO_3_^2–^ (1 000/10 in µmol/L) mixture. Data are derived from panel (A). Insert: Time dependence of parameter ‘b’ calculated from the fitted time-dependent data in the 2–8 min interval shown in panel (B). Arrows mark the addition of SeO_3_^2–^ and GSH into GSNO

As in the case of Cys, we further studied whether the GSH/SeO_3_^2–^ mixture has catalytic properties to release NO from GSNO. Complete NO release was observed after six concesutive additions of 100 µmol/L GSNO to the GSH/SeO_3_^2–^ mixture (Fig. 5A). The detailed kinetics are shown in the Fig. 5B. To compare the rates of NO release after successive GSNO additions parameter ’b’ was used (Fig. 5B, Insert). The release rate was similar for the first three additions of the GSNO, but decreased after the subsequent three additions.

### Comparison of SeO_3_^2–^-Containing Mixtures with Regard to their Ability to Release NO from GSNO

**Fig. 6:**
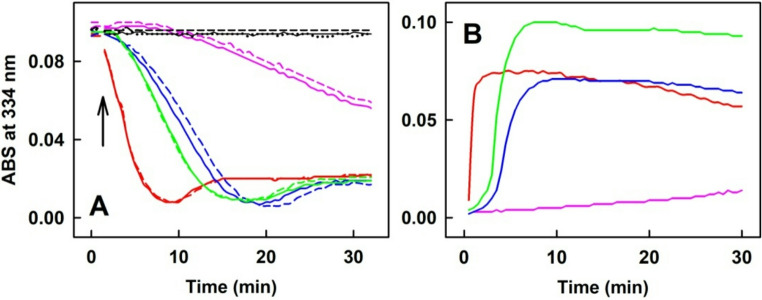
Effects of various compounds on NO release from GSNO. The decrease in ABS at 334 nm indicates the release of NO from GSNO. (**A**) Kinetics of ABS at 334 nm of GSNO/SeO_3_^2–^/compounds (100/10/200 in µmol/L): Cys (red), homocysteine (green), GSH (blue), NAC (pink), GSSG (dotted black), MET (dash black) and cystine (black), *n* = 2. (**B**) Kinetics of ABS at 334 nm of SeO_3_^2–^/compounds (30/200 in µmol/L). Colors correspond to those in panel (A). Absorption spectra were recorded every 30 s for 30 min. The buffer used was 100 mM sodium phosphate, 100 µM DTPA, pH 7.4, 37 °C

The effects of several compounds (200 µmol/L) to release NO from GSNO (100 µmol/L) were compared in the presence of SeO_3_^2–^ (10 µmol/L). The order of potency for NO release was: Cys > HCys > GSH > NAC, whereas cystine, GSSG and MET had no effect (Fig. 6A). The compounds that promoted NO release also reacted with SeO_3_^2–^ (30 µmol/L) without GSNO (Fig. 6B and S8). The rates of increase of ABS at 334, 274, 480 and 700 nm followed the same order as their ability to induce NO release from GSNO, suggesting that product(s) formed during the thiol/SeO_3_^2–^ interaction are responsible for the GSNO decomposition.

### SeMet Interacting with GSH has Catalytic Properties to Release NO from GSNO

**Fig. 7:**
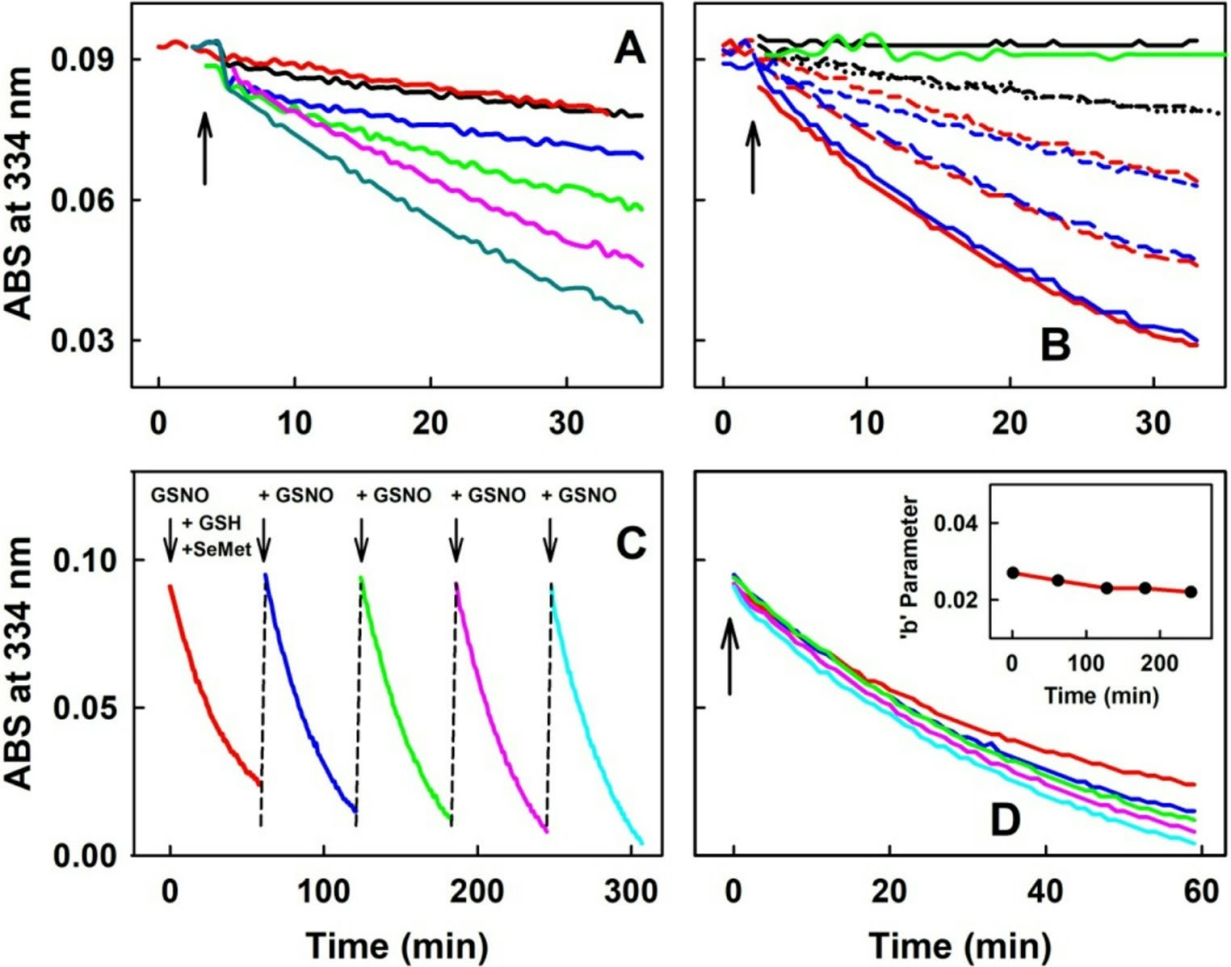
Time-dependence of ABS at 334 nm of UV-Vis spectra during the interaction of SeMet with GSH/GSNO. The decrease in ABS at 334 nm indicates the release of NO from GSNO. (**A**) ABS of 100 µmol/L GSNO with 1 mmol/L GSH without (black) and with 10 (red), 30 (blue), 100 (green), 200 (pink) and 300 (dark cyan) µmol/L SeMet. (**B**) Control ABS of 100 µmol/L GSNO (black) with 2 mmol/L (dash black) and 10 mmol/L (dotted black) GSH. ABS of 100 µmol/L GSNO with 100 µmol/L SeMet (green). ABS of 100 µmol/L GSNO with 5 mmol/L (blue) and 10 mmol/L (red) GSH in the presence of 30 mmol/L (short dash), 100 mmol/L (long dash) and 200 mmol/L (full line) SeMet. UV-Vis absorption spectra were recorded every 30 s for 30 min in 100 mmol/L sodium phosphate aqueous buffer, 100 µmol/L DTPA, pH 7.4, 37 °C. (**C**) Time-dependent effect of 100 µmol/L GSNO in the presence of GSH/SeMet mixture (10/0.1 in mmol/L, red) and followed by four successive additions of 100 µmol/L GSNO (blue, green, pink and cyan). (**D**) Details of the rate of decrease of ABS at 334 nm after five successive additions of GSNO into the GSH/SeMet (10/0.1 in mmol/L) mixture. Data are derived from panel (C). Insert: Time dependence of the parameter ‘b’ calculated from the fitted time-dependent data showed in panel (**C**). Arrows mark the addition of SeMet and GSH to GSNO

GSNO-decomposing effects of SeMet, the most common form of Se found in foods and Se supplements [4], were compared with those of SeO_3_^2–^. SeMet (≥ 30 µmol/L) in the presence of GSH (1, 5 and 10 mmol/L) induced NO release from GSNO in a concentration-dependent manner (Fig. 7A, B). Furthermore, SeMet (≥ 100 µmol/L) in the presence of Cys (200 µmol/L) promoted NO release from GSNO (Fig. S9). The potency of SeMet to induce NO release from GSNO in the presence of GSH or Cys (Figs. 7 and S9) was several times lower than observed with SeO_3_^2–^ (Figs. 2 and 4). Using UV–Vis spectra, we compared the interaction of GSH with SeO3²⁻ and with SeMet (Fig. S9A, B). The UV–Vis spectra of GSH/SeO_3_²⁻ changed over a period of 30 min (Fig. S9A), whereas the UV–Vis spectra of the GSH and SeMet mixture showed no changes during the 30-minute period (Fig. S9B).

As with Cys and GSH, we further studied whether the GSH/SeMet mixture has catalytic properties to promote the release of NO from GSNO. In the presence of excess GSH, repeated additions of 100 µmol/L GSNO (five consecutive times) to the GSH/SeMet mixture resulted in sustained NO release (Fig. 7C). The detailed kinetics are shown in the Fig. 7D. To estimate the rate of the NO release, the parameter ‘b’ was used for comparison (Fig. 7D, Insert) with SeO_3_^2–^. The value of parameter ‘b’ was ~ 0.025 min^–1^, which was several times lower that that observed with SeO_3_^2–^ (Figs. 3 and 5), indicating slower rate of NO release for SeMet compared to SeO_3_^2–^.

### The Effects of Thiols/SeO_3_^2–^ on NO Release from GSNO as Detected by Griess Assay

**Fig. 8:**
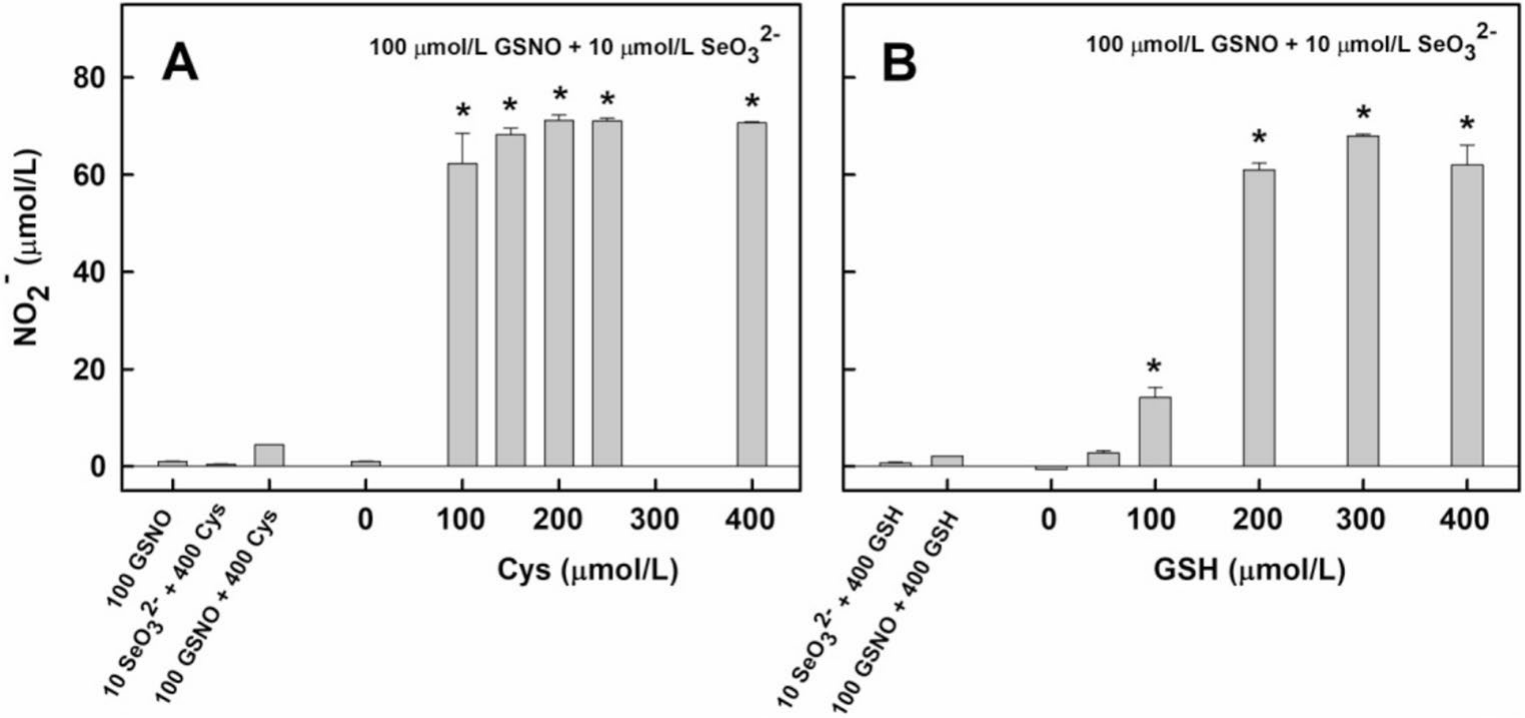
NO release from GSNO as detected by the Griess assay. The increase in NO_2_^–^ indicates the release of NO from GSNO. (**A**) Detection of NO_2_^–^ in the samples: 100 µmol/L GSNO, 10 µmol/L SeO_3_^2–^ with 400 µmol/L Cys, 100 µmol/L GSNO with 400 µmol/L Cys and by the mixture of (100 µmol/L GSNO + 10 µmol/L SeO_3_^2–^) in the presence of 0, 100, 150, 200, 250 and 400 µmol/L Cys. Samples that were significantly different from those with zero concentration of Cys, 100 µmol/L GSNO, 10 µmol/L SeO_3_^2–^ with 400 µmol/L Cys and 100 µmol/L GSNO with 400 µmol/L Cys are marked by *. Data are represented as means ± SD, t-test, *P* < 0.01, *n* = 3. (**B**) Detection of NO_2_^–^ in the samples: 10 µmol/L SeO_3_^2–^ with 400 µmol/L GSH, 100 µmol/L GSNO with 400 µmol/L GSH and by the mixture of (100 µmol/L GSNO + 10 µmol/L SeO_3_^2–^) in the presence of 0, 100, 200, 300 and 400 µmol/L GSH. Samples that were significantly different from those with zero concentration of GSH, 100 µmol/L GSNO, 10 µmol/L SeO_3_^2–^ with 400 µmol/L GSH and 100 µmol/L GSNO with 400 µmol/L GSH are marked by *. Data are represented as means ± SD, t-test, *P* < 0.01, *n* = 3

The Griess reaction was used to confirm that the thiol/SeO_3_^2–^ mixture releases NO from GSNO and that both components, Cys and SeO_3_^2–^ or GSH and SeO_3_²^–^, are required for the release of NO from GSNO and that NO interacted with O_2_ in solution to form the NO oxidation product, nitrite (NO_2_^–^). This approach allowed quantification of NO-derived nitrite and indirect estimation of NO that may have interacted with thiol/SeO_3_^2–^ reaction products. Spontaneous release of NO from GSNO (100 µmol/L) was negligible. Cys or GSH (400 µmol/L) in combination with SeO_3_^2–^ (10 µmol/L) did not interfere with the Griess assay per se. Similarly, Cys or GSH (400 µmol/L) with GSNO (100 µmol/L) induced only minimal NO release. In contrast, the addition of thiols to GSNO/SeO_3_^2–^ mixture significantly increased NO_2_^–^ concentration, reflecting enhanced oxidation of NO released from GSNO (Fig. 8). As ~ 70% of the released NO was detected as NO_2_^–^, it can be assumed that the remaining fraction of NO interacted with products of the thiol/SeO_3_^2–^ reaction.

### Comparison of the Time-Dependent Effect of GSNO and Incubated GSH/SeO_3_^2−^/GSNO Mixture on Rat APW-Ps

Since bioavailability of NO plays a significant role in cardiovascular system [12, 16, 18, 36] and products of the GSH/SeO_3_^2−^ mixture release NO from GSNO in vitro, we compared the effect of GSNO alone and in the incubated GSH/SeO_3_^2−^/GSNO mixture on APW-Ps in SHR rat. In our previous study, IV administration of an incubated GSH/SeO_3_^2−^ mixture (150/25 in mmol/kg) modulated rat APW-Ps [57]. Therefore, in the present study the concentration of the incubated GSH/SeO_3_^2−^ mixture was decreased to 20/2 in mmol/L (corresponding to calculated concentration of GSH/SeO_3_^2–^ in rat 10/1 in µmol/kg) to minimize cardiovascular effects. Using these concentrations, the UV–Vis spectra showed that GSNO did not interact with SeO_3_^2–^; however, after the addition of GSH and 20 s of incubation, the UV–Vis spectra changed, indicating chemical interactions within the mixture after NO release from GSNO (Fig. S9C).

**Fig. 9:**
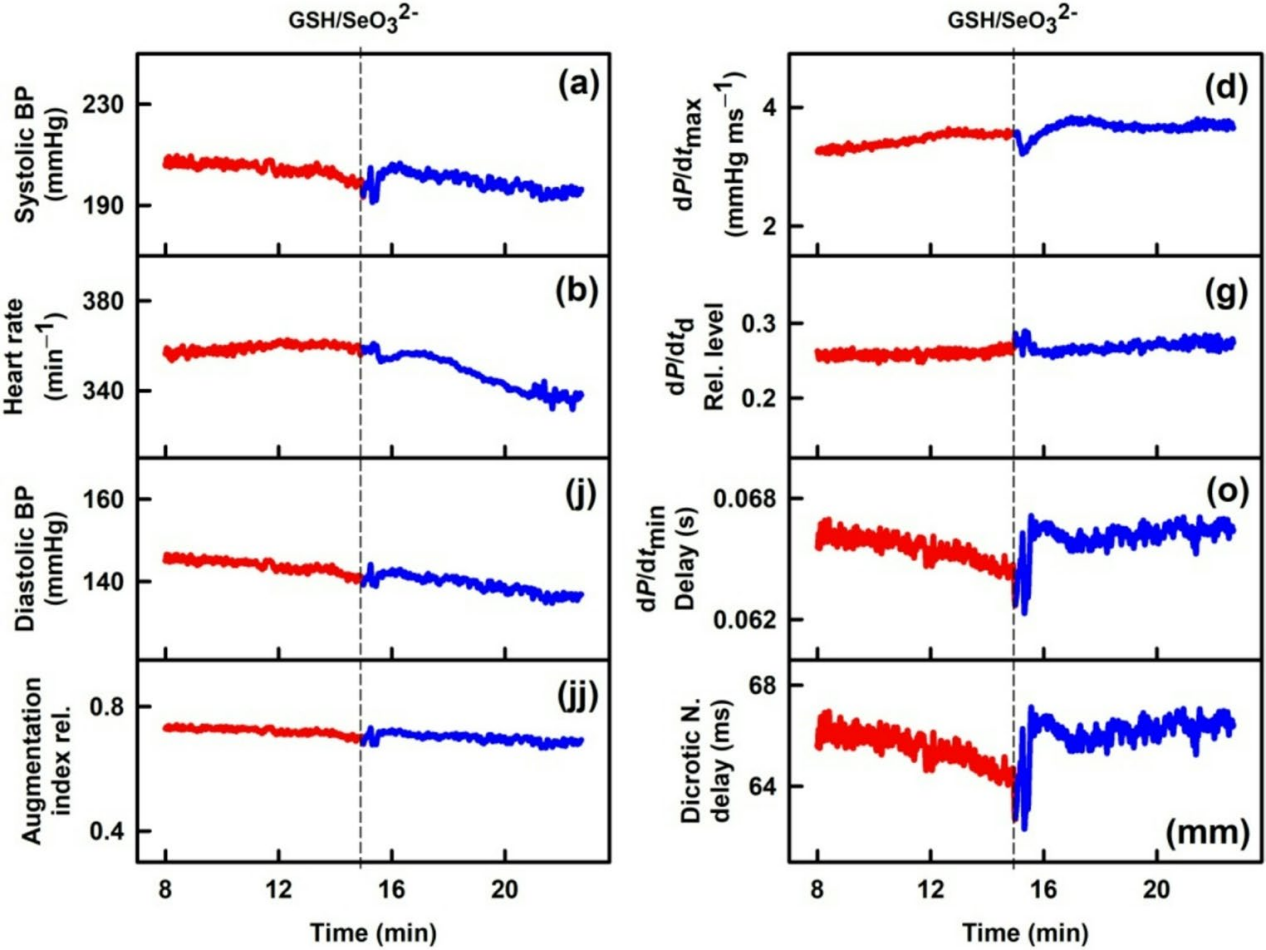
Time-dependent changes of eight APW-Ps at control (red) and after administration of an incubated GSH/SeO_3_^2−^ mixture (10/1 µmol/kg in rat) (blue) in a microtube for 20 s at 23 ± 1 °C. Dashed lines mark the start of mixture administration. Definitions, units and abbreviations of APW-Ps evaluated from the APW are as explained in Supplementary Information Fig. S1

**Fig. 10:**
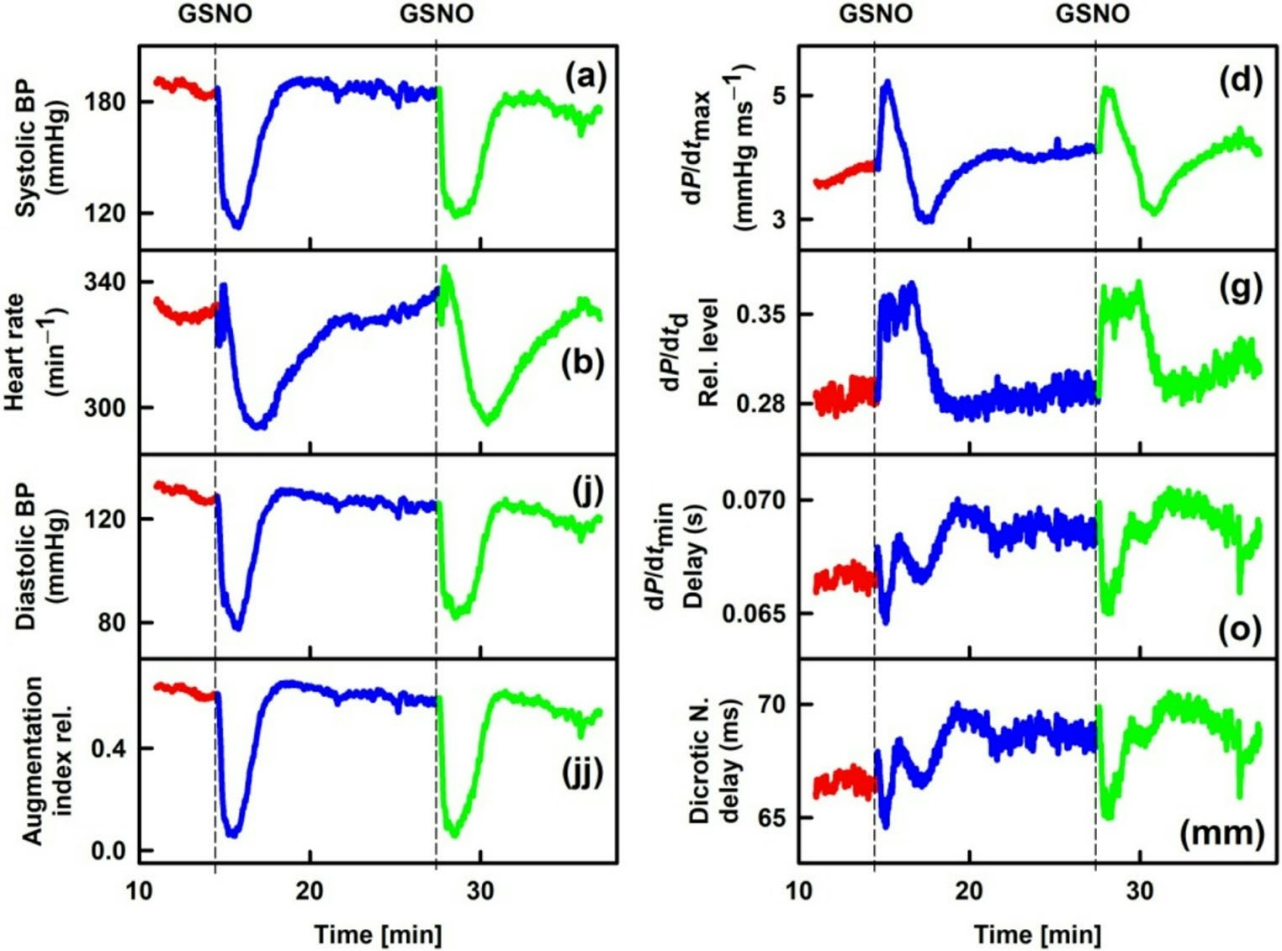
Time-dependent changes in eight APW-Ps under control conditions (red) and after two subsequent administrations of GSNO (80 nmol/kg in rat, blue, green). Dashed lines mark the start of GSNO administration. Definitions, units and abbreviations of APW-Ps evaluated from the APW are as explained in Supplementary Information Fig. S1

**Fig. 11:**
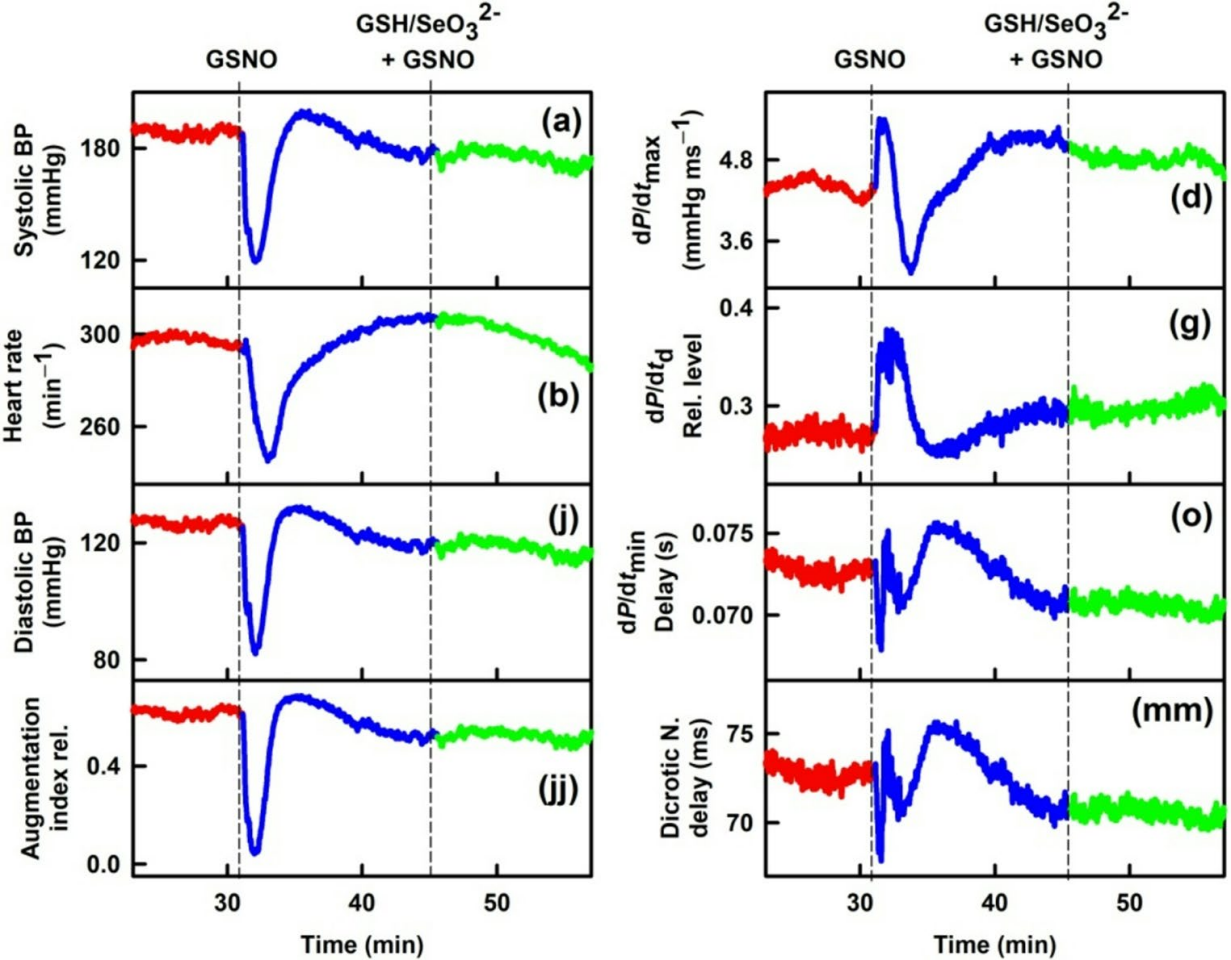
Time-dependent changes of eight APW-Ps under control conditions (red) and after administration of GSNO (80 nmol/kg, blue) and the incubated GSH/SeO_3_^2−^/GSNO mixture (10/1/0.08 µmol/kg in rat, green) in a microtube for 20 s at 23 ± 1 °C. Dashed lines mark the start of compound administration. Definitions, units and abbreviations of APW-Ps evaluated from the APW are as explained in Supplementary Information Fig. S1

In control experiments, IV administration of the incubated GSH/SeO_3_^2−^ (10/1 in µmol/kg in rat) mixture had negligible or minor effect on eight APW-Ps (Fig. 9). In contrast, two consecutive IV administration of GSNO (80 nmol/kg) significantly affected all APW-Ps. GSNO transiently decreased systolic and diastolic BP, heart rate, and augmentation index, transiently increased d*P*/d*t* relative level and produced biphasic effects on several other parameters. These responses were comparable after the first and second GSNO administrations (Fig. 10). When the effects of GSNO alone were compared with those of the incubated GSH/SeO_3_^2−^/GSNO, the mixture had significantly weaker effects on APW-Ps in comparison to the effect of GSNO alone (Figs. 11 and S10–S12).

**Fig. 12:**
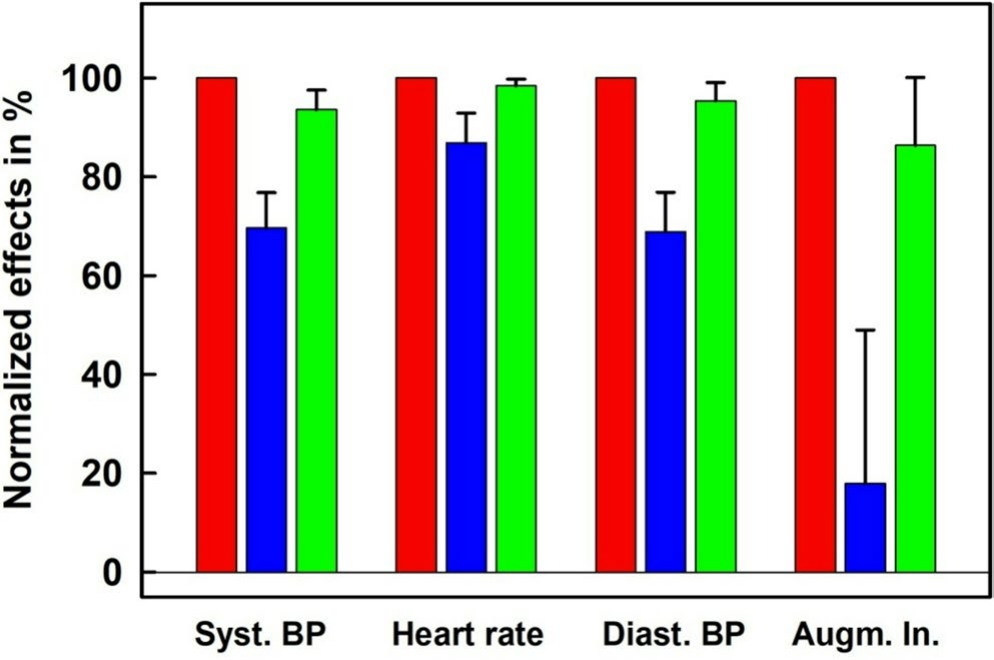
Comparison of the effects of GSNO (80 nmol/kg, blue) and the incubated GSH/SeO_3_^2−^/GSNO mixture (10/1/0.08 µmol/kg in rat, green) on systolic BP (Syst. BP), heart rate, diastolic BP (Diast. BP) and augmentation index (Augm. In.) in SHR rats. Control values (red) were normalized to 100%. Data were derived from Figs. 11 and S10-S12 (*n* = 4). Values are presented as means ± SD. Stastistical significance between effects of GSNO and the incubated GSH/SeO_3_^2−^/GSNO mixture was determined using a t-test, *P* < 0.01 for all comparisons

Since APW-Ps (a), (b), (j) and (jj) showed good reproducibility, whereas APW-Ps (d), (g), (o) and (mm) were less reproducible (Figs. 11 and S10–S12), statistical comparison between GSNO and the incubated GSH/SeO_3_^2−^/GSNO mixture were performed using the four reproducible parameters (Fig. 12). Compared wih GSNO alone, the incubated GSH/SeO_3_^2−^/GSNO mixture produced significantly attenuated responses in all four reproducible APWs.

### SeO_3_^2−^/thiol Interacting with GSNO Induced pDNA Cleavage

**Fig. 13:**
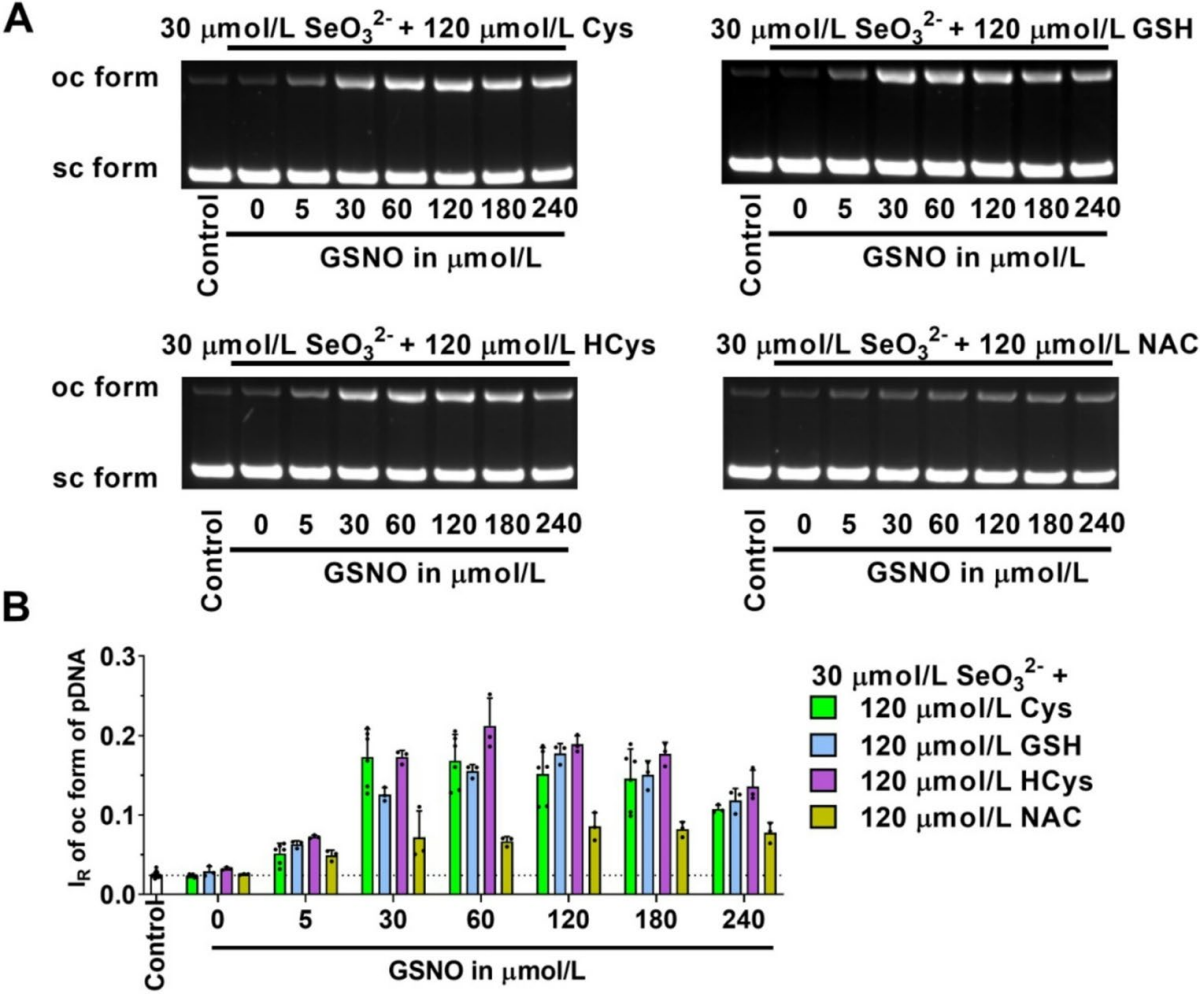
The pDNA cleavage potency of NO released from GSNO induced by thiols/SeO_3_^2–^ mixtures. (**A**) Representative gels showing the effects of increasing concentrations of GSNO (0-240 µmol/L) in the presence of SeO_3_^2–^/Cys (30/120 in µmol/L) or SeO_3_^2–^/GSH (30/120 in µmol/L) on pDNA cleavage. The lower and upper bands correspond to the circular supercoiled (sc) and open circle (oc) forms of pDNA, respectively. The final concentration of pDNA was 0.2 µg in 20 µL of 25 mmol/L sodium phosphate buffer containing 50 µmol/L DTPA, incubated at 37 °C. (**B**) Graphical representation of pDNA cleavage by thiol/SeO_3_^2–^ mixtures in the presence of increasing concentrations of GSNO. The control represents untreated pDNA. I_R_ of oc pDNA form represents the relative intensity of the oc pDNA. Data are presented as individual values and means ± SD (*n* ≥ 3)

Since NO influences DNA cleavage [48, 60, 61] and the SeO_3_^2−^/thiol mixture releases NO from GSNO, we investigated whether NO generated by the SeO_3_^2−^/thiol mixture and/or its derivatives could induce cleavage of pDNA. The pDNA cleavage assay enables detection of reactions leading to the disruption of the sugar-phosphate DNA backbone. In control experiments, SeO_3_^2−^ (30 µmol/L) combined with Cys, HCys, GSH or NAC (120 µmol/L) did not induce pDNA cleavage. GSNO (30–240 µmol/L) in the presence of thiols or SeO_3_^2−^ had only minor or no effects on pDNA integrity (Fig. S13). In contrast, the presence of GSNO (5–240 µmol/L) in the SeO_3_^2−^/thiol mixture resulted in marked pDNA cleavage, displaying a bell-shaped dependance on GSNO concentration (Fig. 13). GSNO concentration, 5 µmol/L in the thiol/SeO_3_^2–^ mixture was sufficient to induce pDNA damage. At maximal effect of the SeO_3_^2−^/thiol/GSNO mixture observed at approximately 60–120 µmol/L GSNO, the cleavage potency followed the order: HCys ≥ GSH ≈ Cys > NAC. To confirm that NO released from GSNO contributed to pDNA damage, the NO scavenger cPTIO was used. cPTIO inhibited pDNA damage induced by the thiol/SeO_3_^2–^/GSNO mixture (Fig. S14). To study whether O_2_^–^ and/or H_2_O_2_ play a role in the mechanism of pDNA damage, SOD which converts superoxide radicals (O₂⁻) into less toxic H_2_O_2_ and CAT, which breaking down H_2_O_2_ into H_2_O and O_2_, were used. Both, SOD or CAT did not protect pDNA damage induced by thiol/SeO_3_^2–^/GSNO mixture (Fig. S14).

## Discussion

**Scheme 1:**
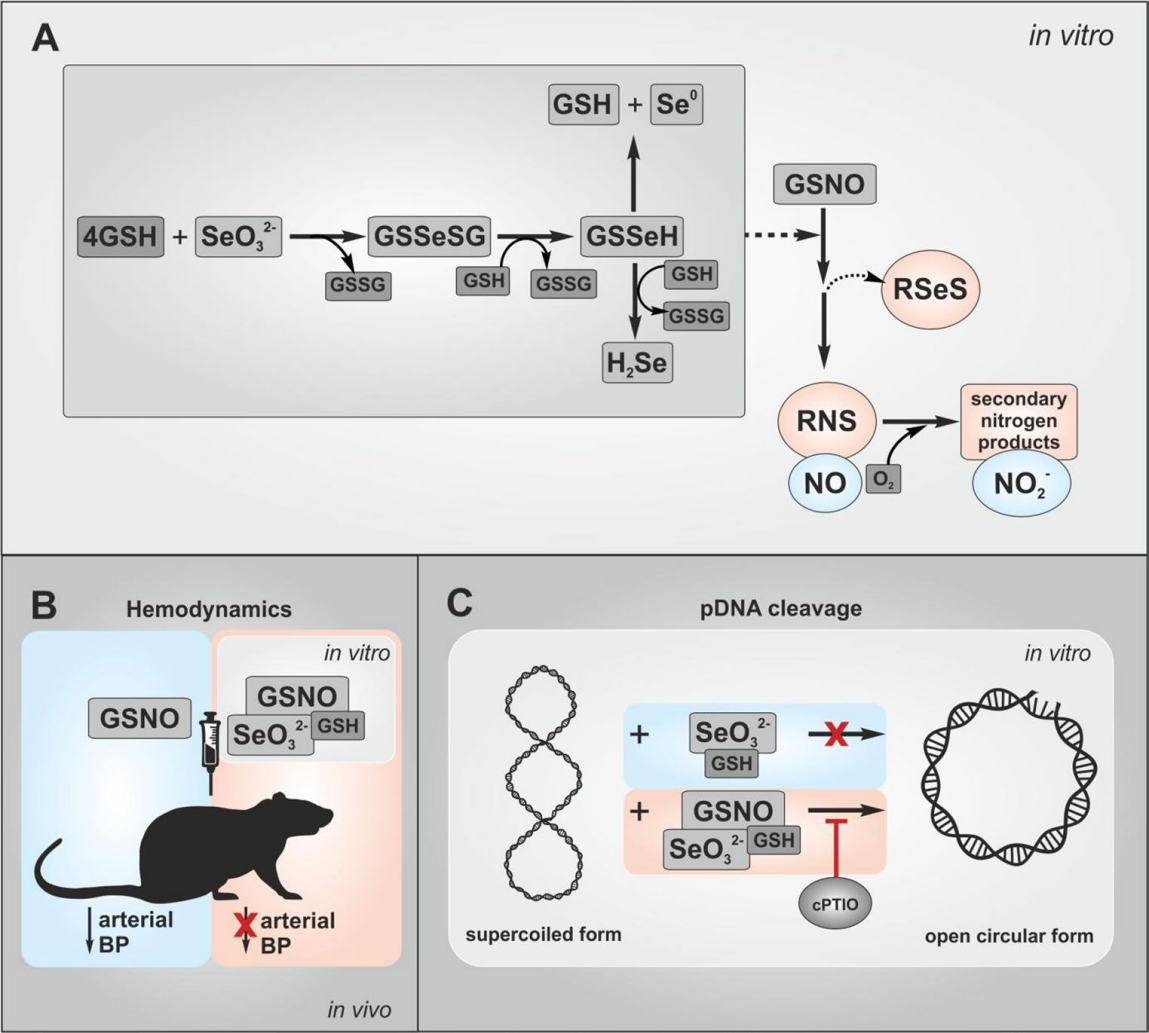
(**A**) Suggested pathway for the decomposition of GSNO in the presence of GSH/SeO₃²⁻ mixture. The reduction of SeO_3_^2–^ by GSH generates different selenium-containing species that likely initiate the decomposition of GSNO, e.g. glutathioselenol (GS–SeH) can act as a nucleophile toward GSNO. Subsequent GSNO decomposition yields reactive nitrogen (RNS) and reactive selenium species (RSeS), including NO, which may be further converted into secondary nitrogen oxides (e.g., NO₂⁻) depending on the prevailing redox conditions. The suggested pathway eliminates free NO in the solution. (**B**) Intravenous administration of GSNO to rats decreases blood pressure. Administration of in vitro prepared GSH/SeO₃²⁻ mixture does not decrease blood pressure, due to that free NO was eliminated in solution by the pathway as suggested in (A). (**C**) Low concentration of GSH/SeO₃²⁻ mixture does not cleavage pDNA. The suggested reactive species produced from the reactions of GSNO/GSH/SeO₃²⁻ mixture cleavage pDNA

The findings that thiol/SeO_3_^2–^ products release NO from GSNO are consistent with previous observations that selenocystamine, selenocystine, organoselenium-derived polymers, and selenium nanoparticles catalyze the decomposition of nitrosothiols in the presence of GSH [3, 37, 38]. Time dependent UV-Vis spectra of the GSNO/thiol/SeO_3_^2–^ mixtures revealed complex reactions that continued even after NO release from GSNO (Figs. 1 and S4, S5). This observation suggests that the released NO may further participate in the secondary reactions leading to reduced NO bioavailability in vivo. The interaction of SeO_3_^2–^ with GSH produces multiple reactive species with distinct biological activities, depending on time and molar ratios [7, 57, 62–65]. SeO_3_^2–^ reacts with GSH to initially form GS-Se-SG. In the presence of excess GSH, GSSeSG is further reduced to GSSeH. GSSeH can either spontaneously dismutate to elemental Se^(0)^ and GSH or undergo further reduction by GSH to yield H_2_Se (see Eqs. 1–14 in [57]). H_2_Se has been implicated in signal transduction pathways and has been suggested as a potential gasotransmitter, analogous to NO, CO and H_2_S [66]. However, H_2_Se is readily oxidized by O_2_ into Se^(0)^ [65, 67]. Based on the qualitatively similar results of NO release from GSNO and the reduction of the stable radical cPTIO by thiol/SeO_3_^2–^ reaction products [57], it can be assumed that the active species responsible for NO release from GSNO are similar to those reducing cPTIO. It is therefore likely that reactive species such as H_2_Se (HSe^–^/Se^2–^) or other negatively charged intermediates, for example RSSe^−^, are involved in the reactions leading to NO release from GSNO (Scheme 1A).

The decrease in NO release rate observed after repeated GSNO additions at lower thiol/SeO_3_^2–^ ratios (parameter ’b’ decreased), most likely reflects progressive thiol depletion and accumulation of oxidized or mixed selenium–derivative species. The catalytic activity requires an excess of reduced thiol to sustain the redox cycle. Notably, 0.3, 0.5 or 1 µmol/L SeO_3_^2–^ in the presence of excess of thiols efficiently induced NO release from 100 µmol/L GSNO, suggesting that these concentrations have catalytic properties on NO release (Figs. 2 and 3). These concentrations of SeO_3_^2–^ are comparable to Se levels in the blood of healthy or hypertensive patients (~ 1 to 3 µmol/L) [68]. It can therefore be assumed that in biological systems, H_2_Se/HSe^–^/Se^2–^ and/or their derivatives may participate in NO release from NO-donors and thus play an important role in regulating NO bioavailability. The results of NO release induced by thiol/SeO₃²⁻ may have consequences for many physiological processes involving the bioavailability of NO [12–22]. It is hypothesized that thiol/SeO₃²⁻ may promote NO release from various endogenous NO donors, thereby contributing to the S-nitrosylation of numerous proteins and cause protein misfolding, thereby contributing to oxidative stress, mitochondrial dysfunction, and cell apoptosis [24–29, 69].

In physiological buffer, GSNO released negligible NO within ~ 10 min (Figs. 8 and S2 [32]), . However, after IV administration of GSNO in rats, the effects of NO release on hemodynamic parameters were observed within a few seconds (Figs. 10 and 11 and S10-S12). We assume that the rapid release of NO from GSNO is the result of GSNO interacting with blood and blood vessel surface components [36]. Such compounds may include Se derivatives. The intracellular concentration of GSH (0.5–10 mmol/L) and Cys (∼200 µmol/L) [70] are sufficiently high to interact with 0.3–10 µmol/L SeO_3_^2−^ or 30–100 µmol/L SeMet, or their derivatives, thereby producing biologically active species capable of releasing NO from endogenous NO donors. The markedly weaker activity of SeMet compared with SeO_3_^2–^ further supports the importance of selenium speciation and redox accessibility. SeO_3_^2–^ is an inorganic, redox-active selenium species that rapidly reacts with thiols to generate reactive intermediates. In contrast, SeMet is a stable selenoether, frequently incorporated nonspecifically into proteins in place of methionine. Negligible reactivity with thiols is confirmed by our UV–Vis study (Fig. S9B), in which SeMet did not interact with GSH. Its mechanism of NO release from GSNO therefore remains unclear.

Several reports demonstrated that SeO_3_^2–^ increases or decreases NO concentrations in various biological systems [39–46, 48], suggesting that this topic is still poorly understood and requires clarification. Similarly, the connections between Se deficiency or excess and various cardiovascular diseases, including its potential myocardial protective role, are not fully understood either [2]. Findings are also controversial regarding the association between the Se concentration and BP [49–54]. A U-shaped relationship between the Se concentrations in blood and cardiovascular mortality rate in hypertensive patients was reported [50], consistent with the concept of Se concentration dependent beneficial and detrimental biological effects. In our study, we found that the GSH/SeO_3_^2−^/GSNO mixture, which had already released NO, exerted only minor effects on APW-Ps in SHR compared with the significant effect of GSNO alone (Figs. 11 and 12 and S10-S12). During the 20 s in-vitro incubation of the GSH/SeO_3_^2–^/GSNO mixture, all NO was released from GSNO and reacted with O_2_ and GSH/SeO_3_^2–^ products (Fig. S9C). Therefore, the incubated mixture had no significant effect on APW-P (Figs. 11 and 12), because it did not contain free NO. Similarly, in the biological system, the thiol/SeO_3_^2–^ mixture can release NO from endogenous NO-donors, and the released NO reacts with biological component before reaching a specific NO-target receptor. We can assume that in the biological system, GSH/SeO_3_^2−^ reduces the bioavailability of NO in situ in this way (Scheme 1B). This is also supported by the in vitro experiments, in which ~ 70% of the released NO was oxidized by O_2_ forming NO_2_^–^ (Fig. 8), while the remaining ~ 30% likely interacted with products of the GSH/SeO_3_^2−^ reaction.

NO is known to damage DNA both in vitro and in vivo [48, 60, 61, 71]. It has been reported to exert both tumor suppressing and promoting effects, depending on its timing, location, and concentration [72]. Polysulfides, mixtures of SeO_3_^2−^ with radicals, or H_2_S with thiols have been found to damage DNA in vitro [57, 59, 73–75]. It is assumed that the mechanisms of NO release and DNA damage are likely interconnected but not identical. NO liberation primarily reflects reductive processes, whereas DNA cleavage is likely mediated by secondary reactive species generated within the same interaction.

Mixtures of thiols/SeO_3_^2−^ have been shown to increase pDNA cleavage [57]. SeO_3_^2−^ oxidizes the majority of the tested thiols in vitro, thereby generating •O_2_^–^ and other ROS [7, 63, 76–78]. It was reported that O_2_^–^ interacting with NO forms peroxynitrite, which can cause pDNA damage [79–81]. In our study we found that NO released from GSNO is involved in pDNA damage (Fig. S14). However, an involvement of O_2_^–^ or H_2_O_2_ in pDNA damage was not confirmed (Fig. S14). The direct in vitro effect of sodium selenide (Na_2_Se) on pDNA has also been studied. It has been found that hydrogen selenide (H_2_Se/HSe^–^/Se^2–^) induced DNA phosphodiester-bond breaks in the presence of O_2_ [73]. Since hydrogen selenide is formed during the thiols/Se_3_^2–^ reaction [57, 82], we suggest that in our study it can also play role in pDNA damage. However, the involvement of other reactive species generated during thiols/Se_3_^2–^/GSNO interaction cannot be excluded (Scheme 1C). The bell-shaped relationship with GSNO concentration suggests that maximal DNA strand break formation occurs at intermediate ratios of NO to thiol/ SeO_3_^2–^ intermediates that may favor formation of free radicals and other reactive species. At higher GSNO concentrations, competing reactions, including radical recombination, or thiol scavenging, may limit the formation of DNA-damaging species.

By comparing the effects of the thiol/SeO_3_^2–^ mixture alone [57] and with GSNO (Fig. 13), it is evident that GSNO amplified the pDNA-damaging effect of thiol/SeO_3_^2–^. This may be relevant to the proposed positive effects of Se in cancer. Since the concentrations of GSH, Cys and HCys vary across different biological compartments and can change under pathological conditions [83–84], we suggest that the NO-releasing and pDNA-damaging effects of thiol/SeO_3_^2−^/GSNO depend on the local concentrations of GSH, Cys or HCys and on their thiol/disulfide redox couples in situ.

Our findings contribute to the understanding of the diverse and sometimes controversial effects of selenium compounds. They suggest that selenium supplements, particularly selenite, can significantly affect the bioavailability of NO, which can influence many NO-dependent biological functions. They may promote the formation of reactive intermediates that, under certain conditions, may contribute to redox imbalance or nitrosative stress, which can affect the cardiovascular system and DNA.

### Limitations

A limitation of this study is the insufficient understanding of the individual chemical reactions and substances responsible for the release of NO from GSNO, the molecular mechanism of pDNA damage, and the modulation of cardiovascular parameters. As a result, we cannot definitively determine which effects are caused by redox reactions and which are mediated by specific selenium-containing intermediates. Further studies using advanced analytical techniques will be necessary to clarify the exact chemical nature of the active substances involved in this process. This remains a challenge for future studies.

### In Conclusion

Our study provides evidence that thiol/SeO_3_^2–^ interactions with NO-donors influence NO bioavailability and signaling, thereby affecting both cardiovascular system and DNA stability. Specifically, these interactions induced the release of NO from GSNO, but paradoxically reduced GSNO-mediated effects on rat cardiovascular parameters. Moreover, GSNO promoted pDNA cleavage in the presence of the GSH/SeO_3_^2–^ mixture. We assume that part of the released NO from GSNO subsequently reacts with some of the GSH/ SeO_3_^2–^ products, resulting in decreased NO bioavaibility in situ and the formation of species that disrupt DNA stability. We hypothesize that Se’s dual roles in cardiovascular regulation and cancer prevention may be, at least in part, due to its ability to modulate NO-related processes.

## Supplementary Information

Below is the link to the electronic supplementary material.

Supplementary Material 1 (PDF 1.53 MB)

## Data Availability

All findings and conclusions are based on the presented figures in the main text or in the Supplementary Materials. Original source files can be sent from the corresponding author, Dr. Karol Ondrias, upon request.
