# Supplementary material for: Selenite Interacting with Thiols Catalytically Releases NO from GSNO, Cleaves Plasmid DNA and Reduces the Hypotensive Effect of GSNO in Rats
Source: Biol Trace Elem Res. 2026 Apr 14;204(8):6205–22. doi: 10.1007/s12011-026-05105-6 (PMC13368853; doi:10.1007/s12011-026-05105-6)
Supplement: Supplementary file 1 — Supplementary Material 1 (PDF 1.53 MB) [file 12011_2026_5105_MOESM1_ESM.pdf]

Supplementary Information

to

Selenite interacting with thiols catalytically releases NO from GSNO, cleaves plasmid DNA and reduces the hypotensive effect of GSNO in rats

Marian Grman [1], Anton Misak [1], Lenka Tomasova [1], Miroslav Chovanec [2], Karol Ondrias [1*]

[1] Institute of Clinical and Translational Research, Biomedical Research Center, Slovak Academy of Sciences, Dubravska cesta 9, 845 05 Bratislava, Slovak Republic; marian.grman@savba.sk (M.G.); anton.misak@savba.sk (A.M.); lenka.tomasova@savba.sk (L.T.); karol.ondrias@savba.sk (K.O.)
[2] Cancer Research Institute, Biomedical Research Center, Slovak Academy of Sciences, Dubravska cesta 9, 845 05 Bratislava, Slovak Republic; miroslav.chovanec@savba.sk (M.C.)

* Correspondence: karol.ondrias@savba.sk

Description of eight parameters (APW-Ps) from APW:

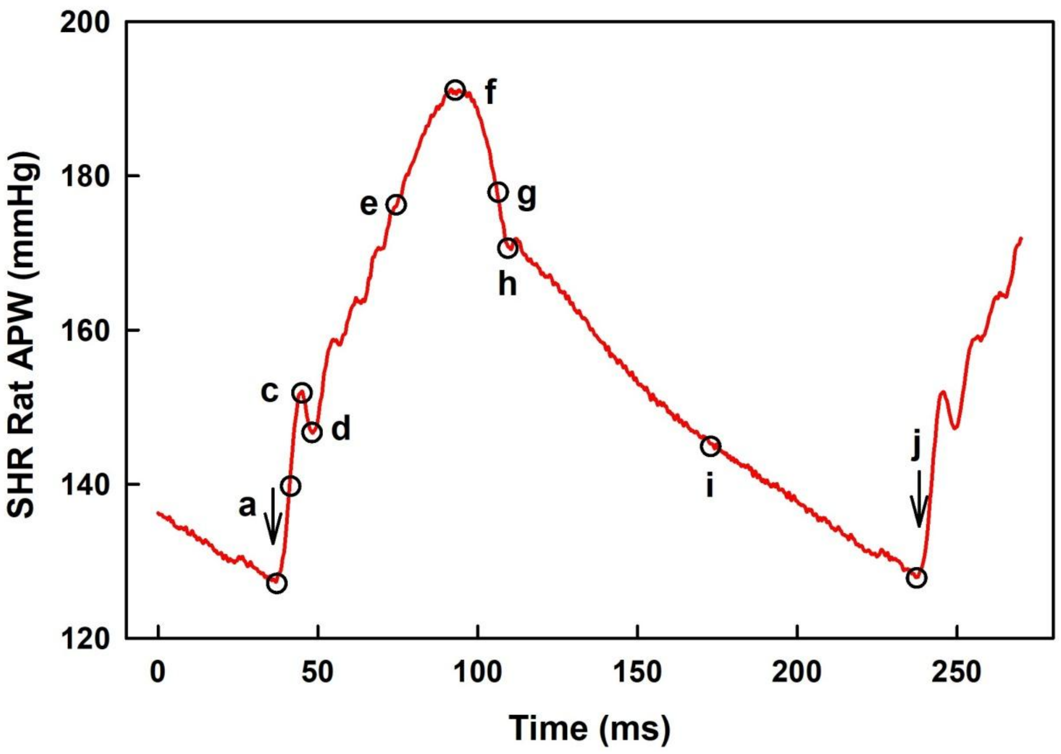

Fig. S1 The left common carotid artery pulse waveform (APW) in the anesthetized SHR rat with marked ten points **a** - **j** (black circles)

Ten points **a - j** (in bold letters) are from Figure S1 and they mark the values of BP and time that are used to define (calculate) specific APW-Ps.

APW parameters:

(a) Systolic blood pressure in mmHg; point **c** or **f**.
(b) Heart rate in min^{-1}; 60 / (**j** – **a**); (**j** – **a**) represents time interval between **a** and **j**, **a** and **j** are two reference points to diastolic BP value.
(j) Diastolic blood pressure in mmHg; the point **a** or **j.**
(jj) Augmentation index relative; (**f** – **c**) / (**f** – **a**) in mmHg/mmHg (dimensionless)*.
(d) dP/dt_{max} in mmHg ms^{-1}; maximum derivative at the point **b**; P is BP in mmHg.
(g) dP/dt_d relative level, relative level of point **i**; (**i** – **a**) / (**c** (or **f**) – **a**) in mmHg/mmHg (dimensionless).
(o) dP/dt_{min} delay in s; delay in s of point **g**; (**g** – **a**) time interval between **a** and **g**.
(mm) Dicrotic notch delay in ms, delay in ms of point **h**; (**h** – **a**) time interval between **a** and **h**.

*The plot of augmentation index relative (**jj**) was not possible to determine in cases when the highest point at APW (Fig. S1) was "c" and not "f" and it was set to zero (Kurakova *et al*., 2020; Tomasova *et al*., 2021).

Figures:

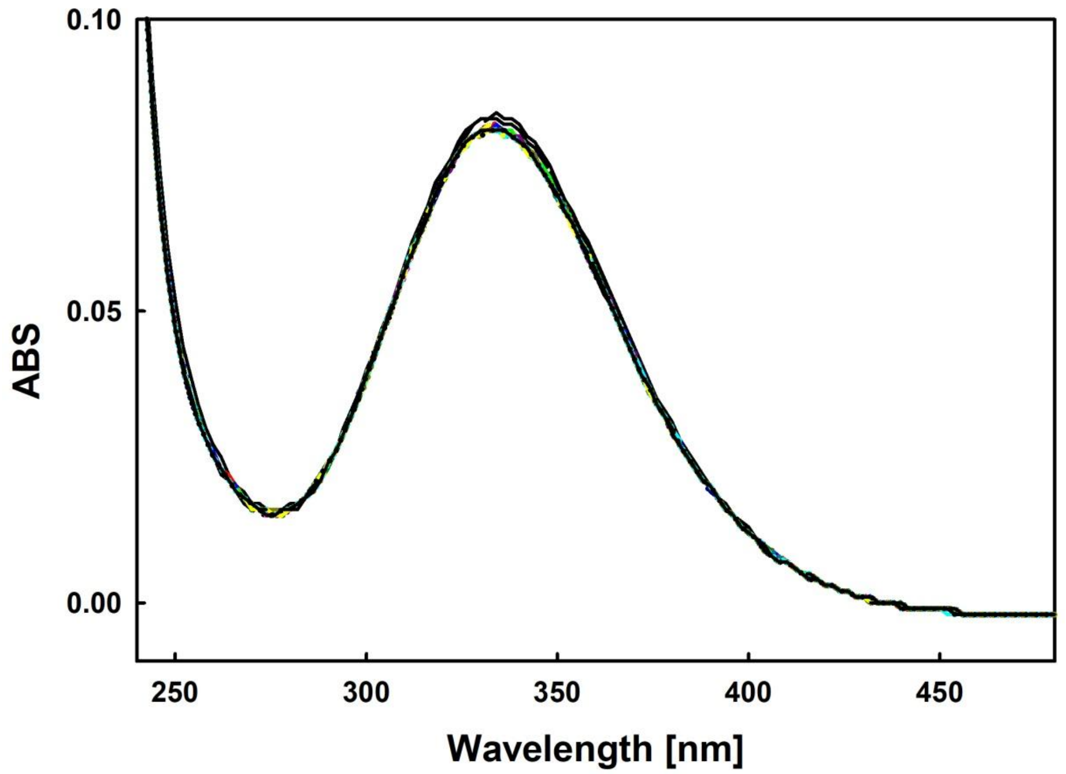

Fig. S2 Representative time resolved UV-Vis spectra of the interaction of SeO_3^{2-} with GSNO. UV-Vis spectra of GSNO/SeO_3^{2-} (100/100 in µmol/L, final) mixture was measured every 30 s for 30 min in 100 mmol L^{-1} sodium phosphate, 100 µmol/L DTPA, pH 7.4, 37°C. The decrease in ABS at 334 nm indicates the release of NO from GSNO

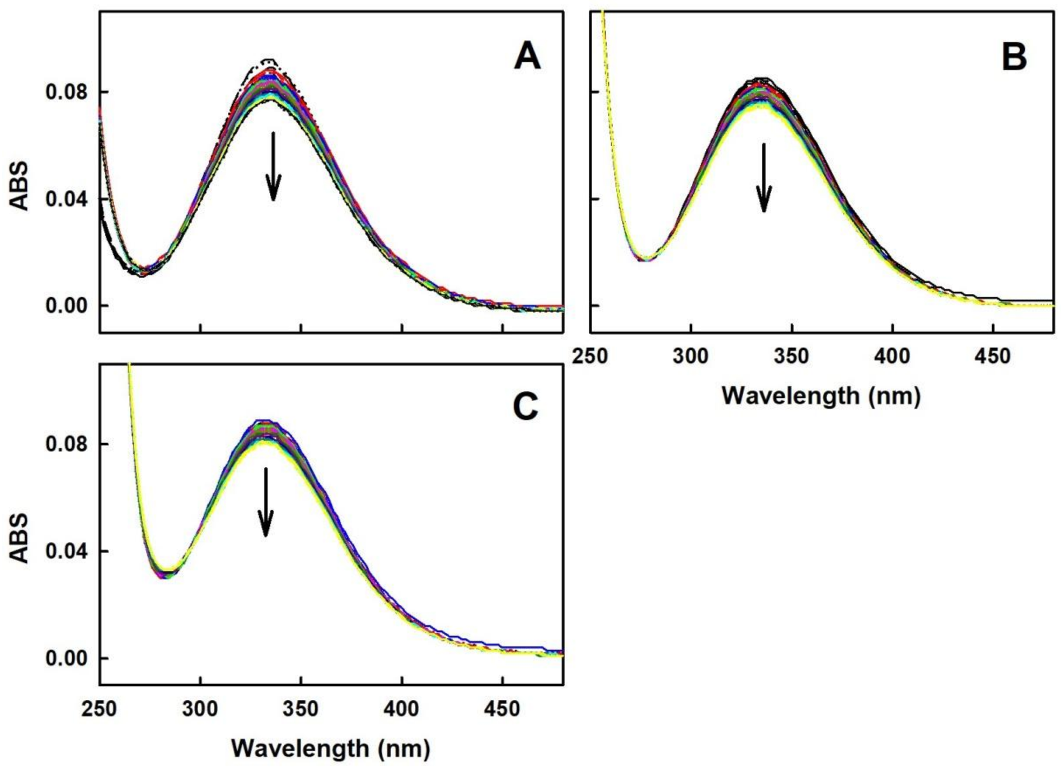

Fig. S3 Representative time-resolved UV-Vis spectra of the interaction of Cys or GSH with GSNO. UV-Vis spectra of GSNO/Cys (100/200 in μmol/L) (A), (100/1 000 in μmol/L) (B) and GSNO/GSH (100/10 000 in μmol/L) (C). The mixture was measured every 30 s for 30 min in 100 mmol/L sodium phosphate, 100 μmol/L DTPA, pH 7.4, 37°C. Arrows show direction of NO release from GSNO. The decrease in ABS at 334 nm indicates the release of NO from GSNO

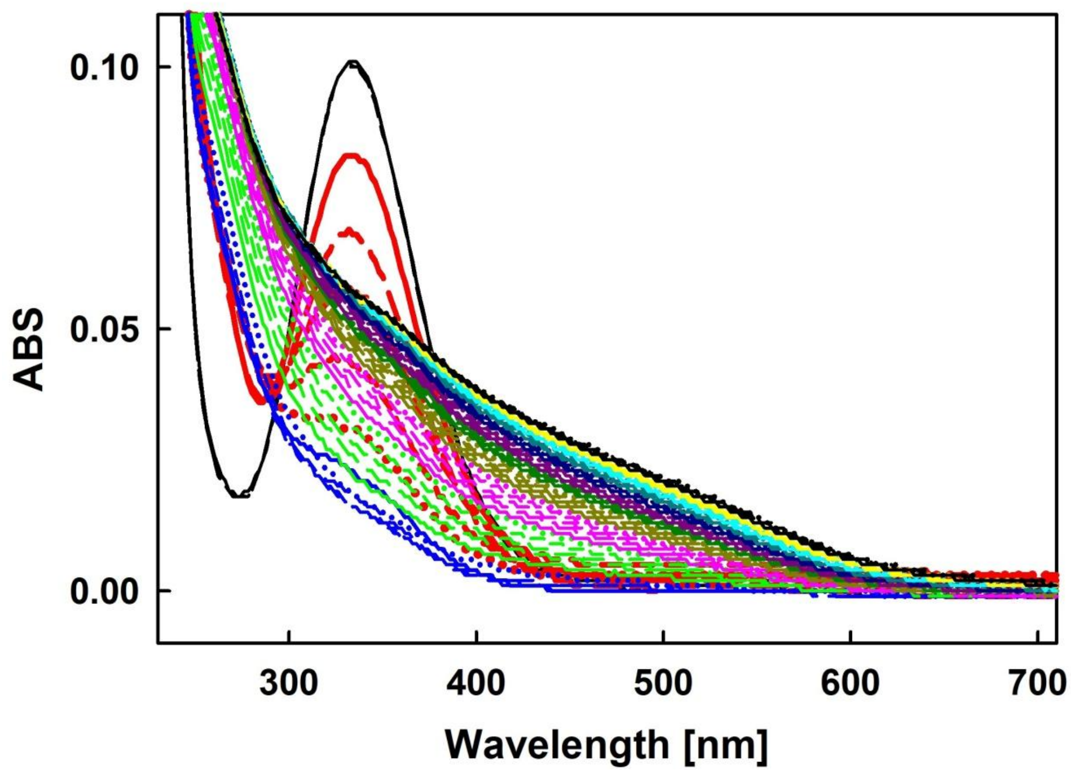

Fig. S4 Representative time-resolved UV-Vis spectra of the interaction of SeO_3^{2-} with GSNO in the presence of Cys. UV-Vis spectra of GSNO (100 μmol/L, final) was measured every 30 s for 1.5 min in 100 mmol/L sodium phosphate, 100 μmol/L DTPA, pH 7.4, 37°C (control black lines) followed by subsequent addition of SeO_3^{2-} (30 μmol/L, final) and Cys (200 μmol/L, final) 15 s later and measured every 30 s for 30 min. The solid red line indicates the first spectrum after addition of SeO_3^{2-}/Cys, which is followed each 30 s by: long dash red, medium dash red, short dash red, dotted red, solid blue line, long dash blue, medium dash blue, *etc*. The decrease in ABS at 334 nm indicates the release of NO from GSNO

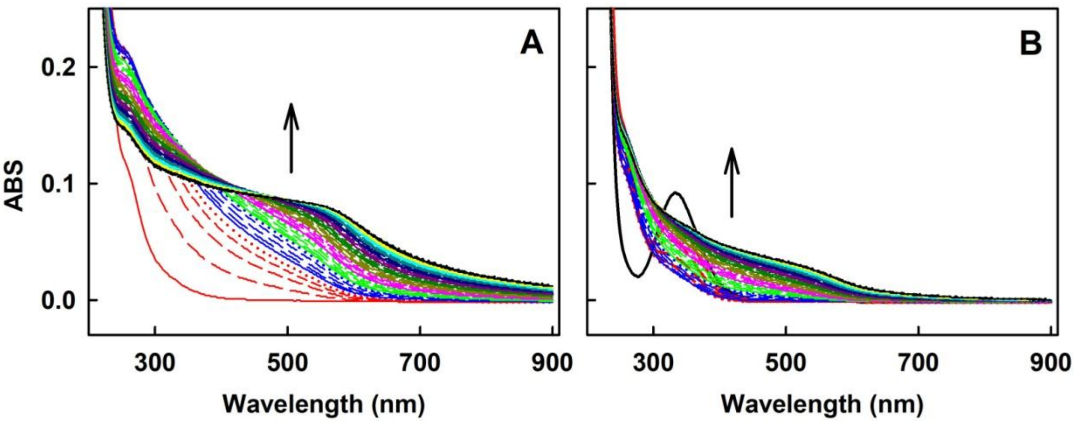

Fig. S5 Representative time-resolved UV-Vis spectra of the interaction of SeO_3^{2-} with Cys. (A) Time resolved UV-Vis of SeO_3^{2-}/Cys (100/200 in μmol/L, final) mixture measured every 30 s for 30 min. (B) UV-Vis spectrum of GSNO (100 μmol/L, final) in 100 mmol/L sodium phosphate, 100 μmol/L DTPA, pH 7.4, 37°C (black) followed by subsequent addition of SeO_3^{2-} (100 μmol/L, final) and Cys (200 μmol/L, final) 15 s later and measured every 30 s for 30 min The solid red line indicates the first spectrum after addition of SeO_3^{2-}/Cys, which is followed each 30 s by: long dash red, medium dash red, short dash red, dotted red, solid blue line, long dash blue, medium dash blue, *etc*. The decrease in ABS at 334 nm indicates the release of NO from GSNO

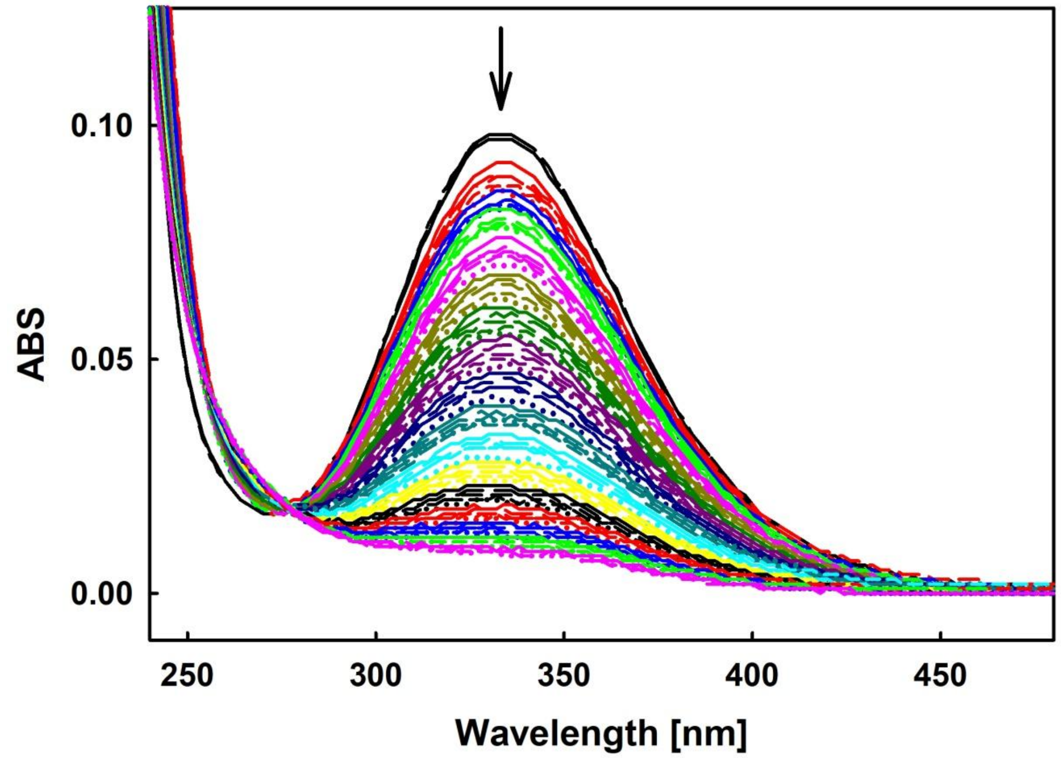

Fig. S6 Representative time-resolved UV-Vis spectra of the interaction of SeO_3^{2-} with GSNO/Cys. UV-Vis spectra of GSNO (100 μmol/L, final) was measured every 30 s for 1.5 min in 100 mmol/L sodium phosphate, 100 μmol/L DTPA, pH 7.4, 37°C (black lines) followed by subsequent addition of SeO_3^{2-} (0.5 μmol/L, final) and Cys (200 μmol/L, final) and measured every 30 s for 43 min. The solid red line indicates the first spectrum after addition of SeO_3^{2-}/Cys, which is followed each 30 s by: long dash red, medium dash red, short dash red, dotted red, solid blue line, long dash blue, medium dash blue, *etc*. Decrease of ABS at 334 nm indicates NO release from GSNO (arrow). The decrease in ABS at 334 nm indicates the release of NO from GSNO

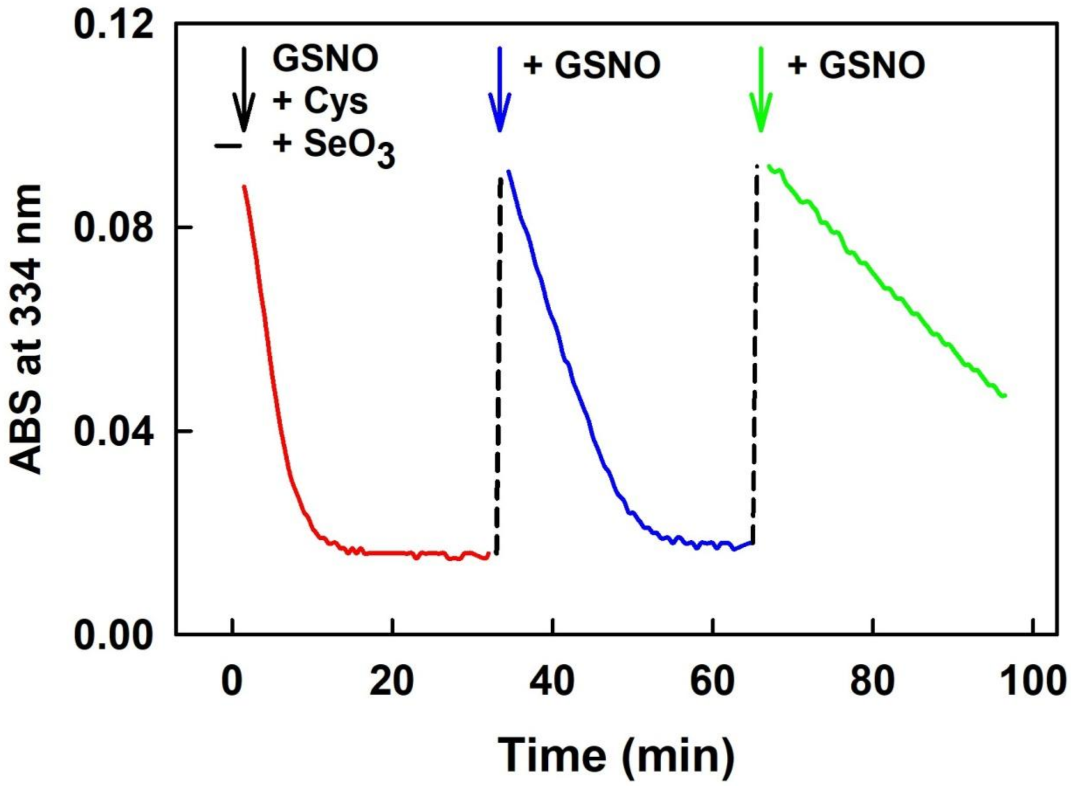

Fig. S7 Time dependence of ABS at 334 nm of UV-Vis spectra during the interaction of SeO_3^{2-} with GSNO/Cys. UV-Vis spectra of GSNO (100 μmol/L, final) was measured every 30 s for 1.5 min in 100 mmol/L sodium phosphate, 100 μmol/L DTPA, pH 7.4, 37°C (full black) followed by subsequent addition of SeO_3^{2-} (3 μmol/L, final) and Cys (200 μmol/L, final) 15 s later (black arrow) and the spectra were measured every 30 s for 30 min (red). Then GSNO (100 μmol/L, final) was added into the mixture (blue arrow) and the spectra were measured every 30 s for 30 min (blue). Then GSNO (100 μmol/L, final) was added again (green arrow) into the mixture and the spectra were measured every 30 s for 30 min (green). The decrease in ABS at 334 nm indicates the release of NO from GSNO

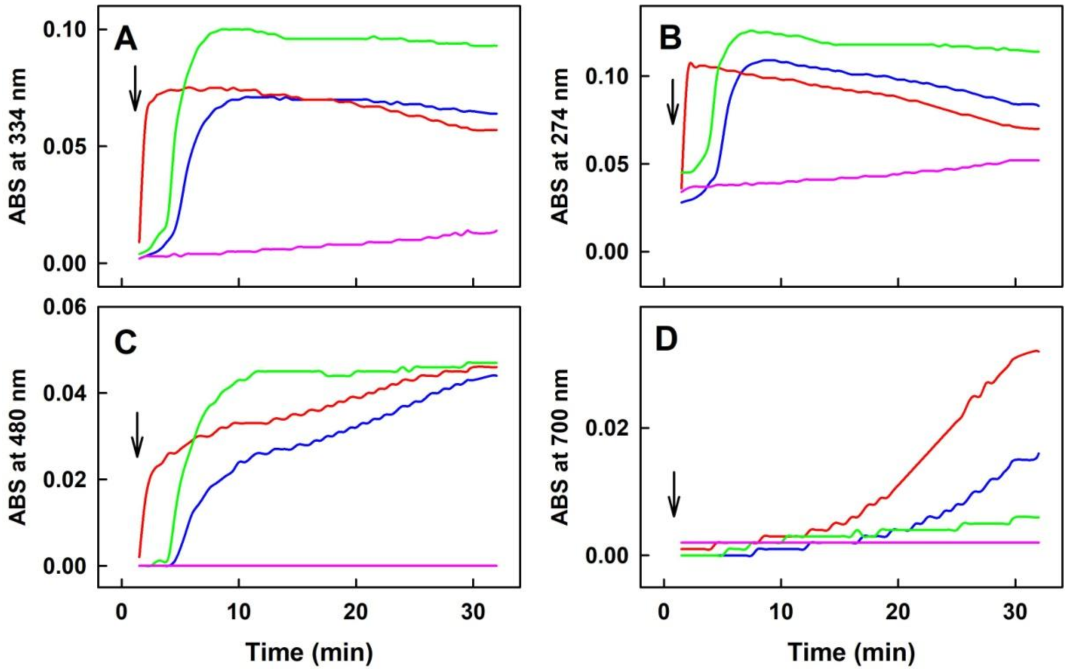

Fig. S8 Time dependent of ABS at 334 (A), 274 (B),, 480 (C) and 700 nm (D) of UV-Vis spectra during the interaction of 30 μmol/L SeO_3^{2-} with 200 μmol/L of Cys (red), GSH (blue), HCys (green) and NAC (pink)

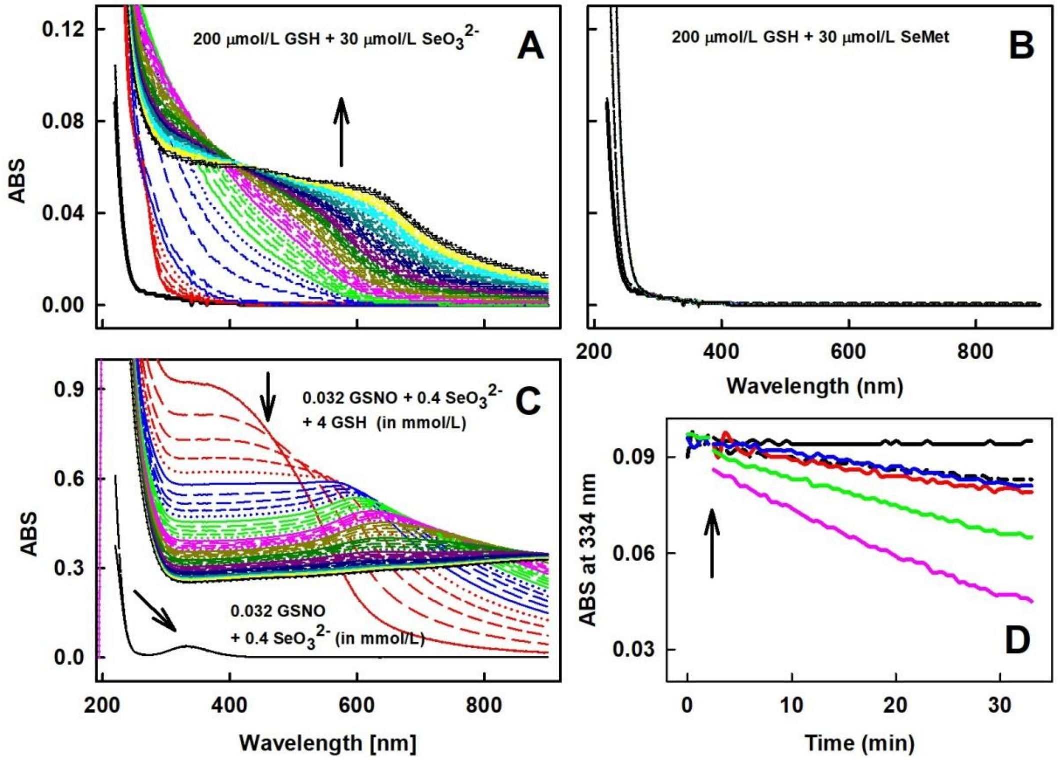

Fig. S9.

(A) Representative time-resolved UV-Vis spectra of the interaction of SeO_3^{2-} with GSH. UV-Vis spectra of SeO_3^{2-} (30 μmol/L, final) was measured every 30 s for 2.5 min in 100 mmol/L sodium phosphate, 100 μmol/L DTPA, pH 7.4, 37°C (black lines) followed by subsequent addition of GSH (200 μmol/L, final) and measured every 30 s for 30 min. The solid red line indicates the first spectrum after addition of GSH, which is followed each 30 s by: long dash red, medium dash red, short dash red, dotted red, solid blue line, long dash blue, medium dash blue, *etc*.

(B) Representative time-resolved UV-Vis spectra of the interaction of SeMet with GSH. UV-Vis spectra of SeMet (30 μmol/L, final) was measured every 30 s for 2.5 min in 100 mmol/L sodium phosphate, 100 μmol/L DTPA, pH 7.4, 37°C (black lines) followed by subsequent addition of GSH (200 μmol/L, final) and measured every 30 s for 30 min. The UV-Vis spectra did not change for 30 min.

(C) Representative time-resolved UV-Vis spectra of the interaction of GSNO/SeO_3^{2-} with GSH. UV-Vis spectra of GSNO (32 μmol/L, final) without and with SeO_3^{2-} (400 μmol/L, final) was measured every 30 s for 2.5 min in 100 mmol/L sodium phosphate, 100 μmol/L DTPA, pH 7.4, 37°C (black lines) followed by subsequent addition of GSH (4000 μmol/L, final) and measured every 30 s for 30 min. The solid red line indicates the first spectrum after addition of GSH, which was measured 20 s after GSH addition. It is followed each 30 s by: long dash red, medium dash red, short dash red, dotted red, solid blue line, long dash blue, medium dash blue, *etc*.

(D) Time dependence of ABS at 334 nm of UV-Vis spectra during the interaction of SeMet with GSNO/Cys. ABS of 100 μmol/L GSNO without (black) and with 200 μmol/L Cys (dash black). The mixture GSNO/Cys (100/200 in μmol/L) after addition of 10 (red), 30 (blue), 100 (green) and 200 μmol/L (pink) SeMet. UV-Vis spectra were measured every 30 s for 30 min in 100 mmol/L sodium phosphate, 100 μmol/L DTPA, pH 7.4, 37°C

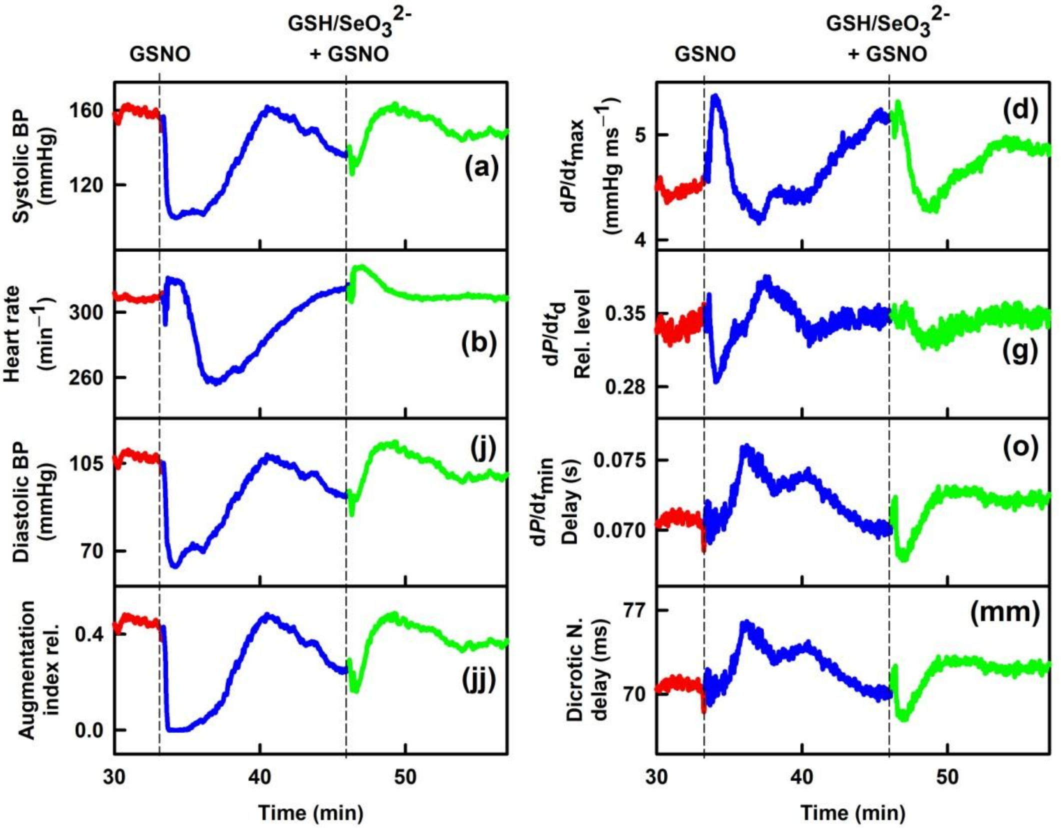

Fig. S10 Time-dependent changes of eight APW-Ps at control (red) and after administration of GSNO (80 nmol/kg, blue) and incubated GSH/SeO_3^{2-}/GSNO (10/1/0.08 μmol/kg in rat, green) mixture in a microtube for 20 s at 23±1°C. Dashed lines mark the start time of compounds administration. Definitions, units and abbreviations of APW-Ps evaluated from the APW are as explained in Supplementary Information Fig. S1

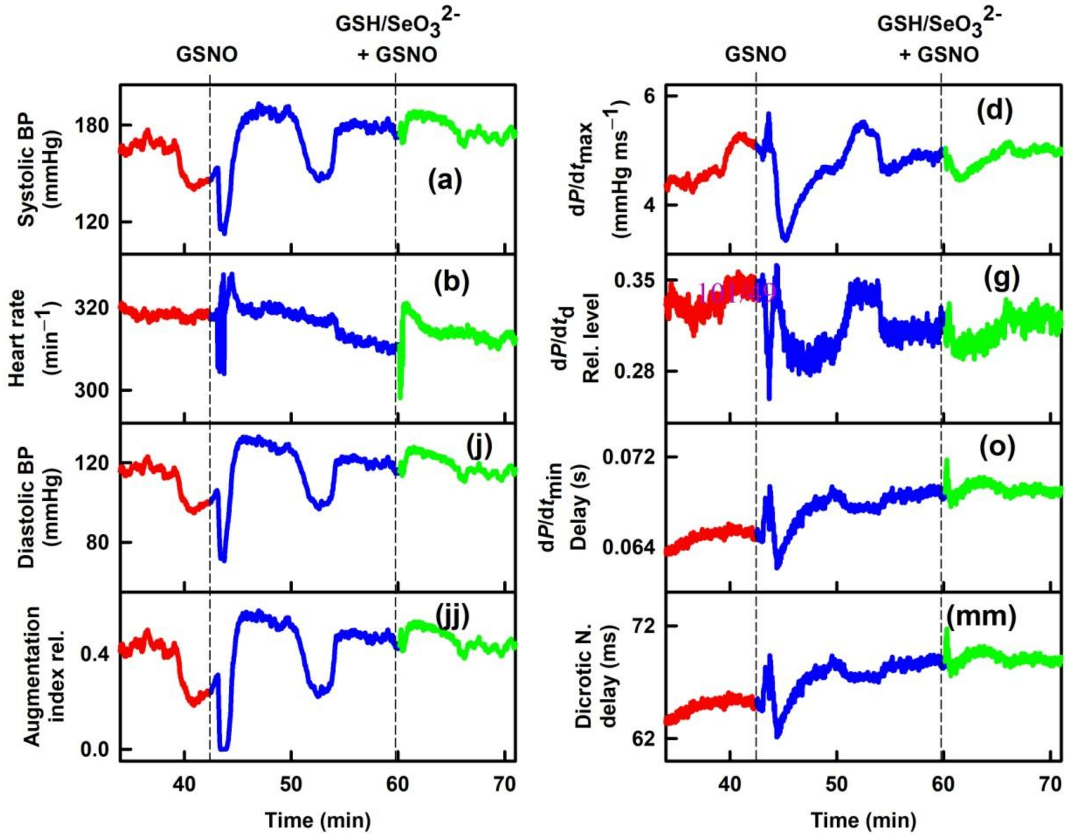

Fig. S11 Time-dependent changes of eight APW-Ps at control (red) and after administration of GSNO (80 nmol/kg, blue) and incubated GSH/SeO_3^{2-}/GSNO (10/1/0.08 μmol/kg in rat, green) mixture in a microtube for 20 s at 23±1°C. Dashed lines mark the start time of compounds administration. Definitions, units and abbreviations of APW-Ps evaluated from the APW are as explained in Supplementary Information Fig. S1

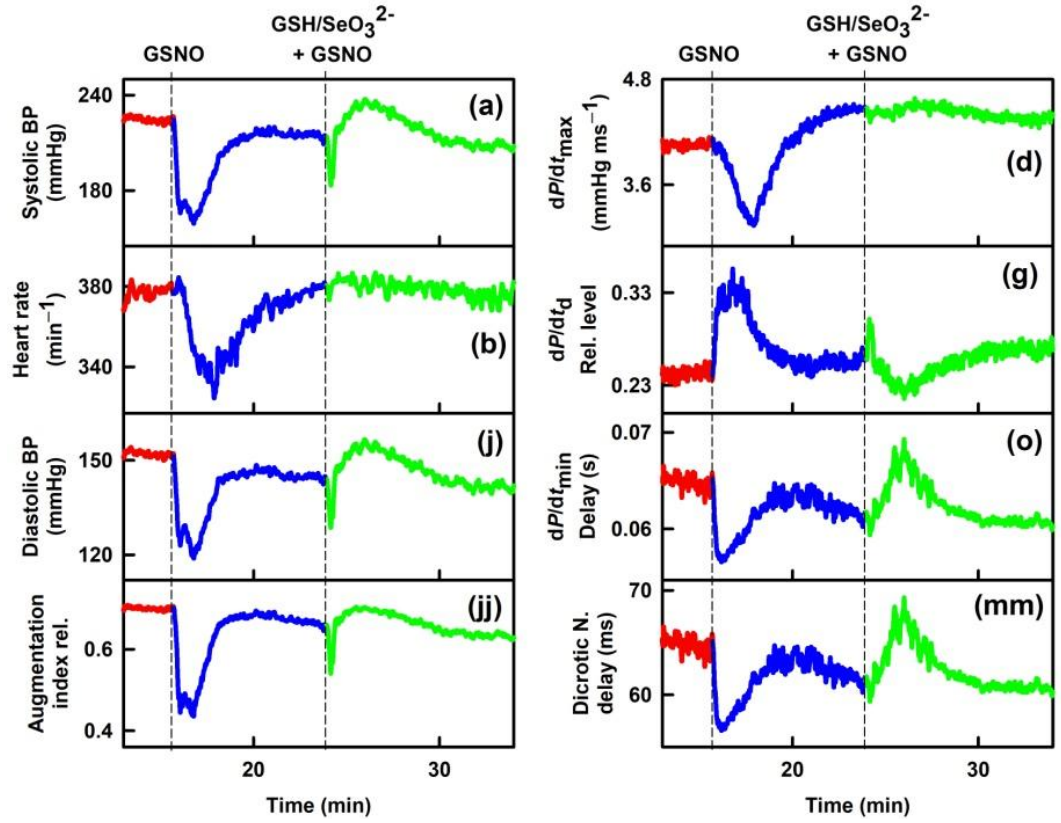

Fig. S12 Time-dependent changes of eight APW-Ps at control (red) and after administration of GSNO (80 nmol/kg, blue) and incubated GSH/SeO_3^{2-}/GSNO (10/1/0.08 μmol/kg in rat, green) mixture in a microtube for 20 s at 23±1°C. Dashed lines mark the start time of compounds administration. Definitions, units and abbreviations of APW-Ps evaluated from the APW are as explained in Supplementary Information Fig. S1

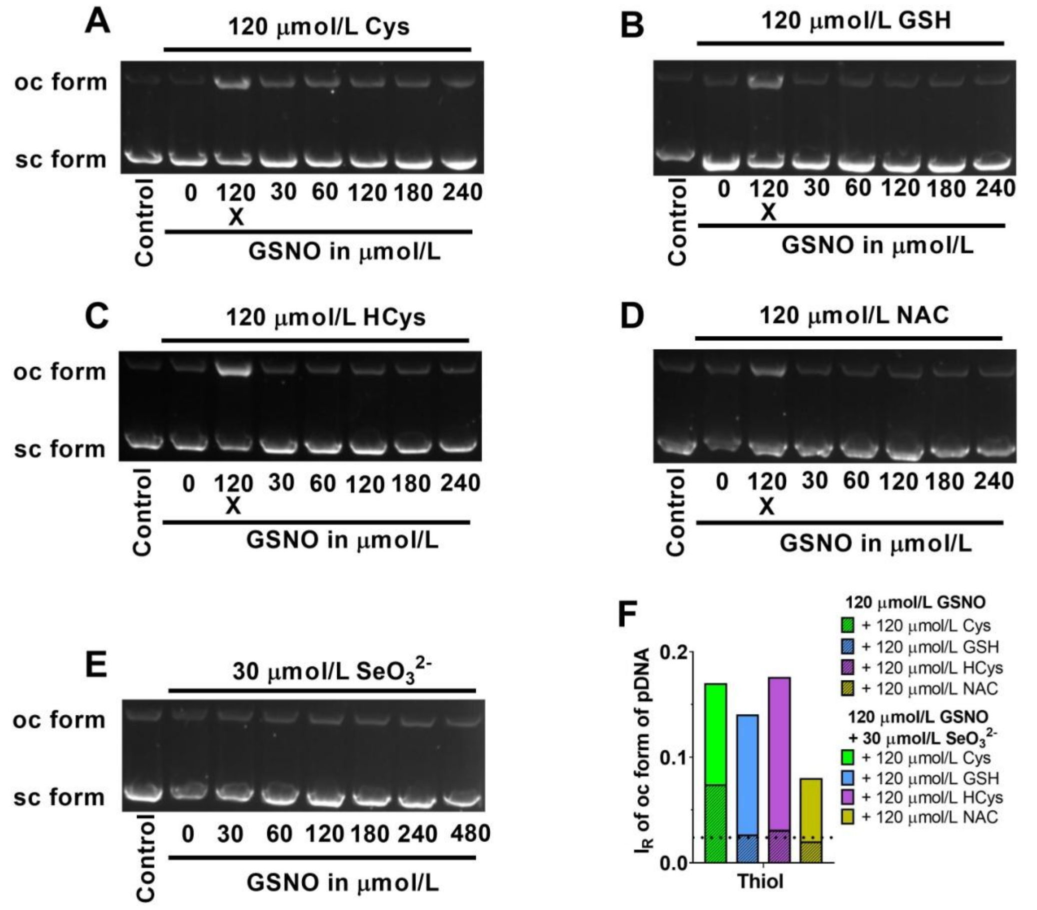

Fig. S13 Effect of thiols on pDNA cleavage potency of GSNO in the absence of SeO_3^{2-}. Representative gels indicating the pDNA cleavage effects of increasing concentrations of GSNO (0 – 240 μmol/L) in the presence of 120 μmol/L Cys (A), GSH (B), HCys (C), or NAC (D). The pDNA cleavage (detected as the open circle form of pDNA) was minor, if any, when compared to that generated due to the presence of 30 μmol/L SeO_3^{2-} in the reaction (indicated with X mark). The reaction of 30 μmol/L SeO_3^{2-} with GSNO (0 – 480 μmol/L) did not cleave pDNA (E). The bands at the bottom and top correspond to the circular supercoiled (sc) and open circle (oc) forms of pDNA, respectively. The quantification of pDNA cleavage induced by 120 μmol/L thiol and 120 μmol/L GSNO in the presence (superimposed, without filling) or absence of 30 μmol/L SeO_3^{2-} (hatched) (F). A dotted line indicates control experiment without any treatment. Data are taken from A-D (*n* = 1). The final concentration of pDNA was 0.2 μg in 20 μL in a 25 mmol/L sodium phosphate buffer and 50 μmol/L DTPA at 37 °C

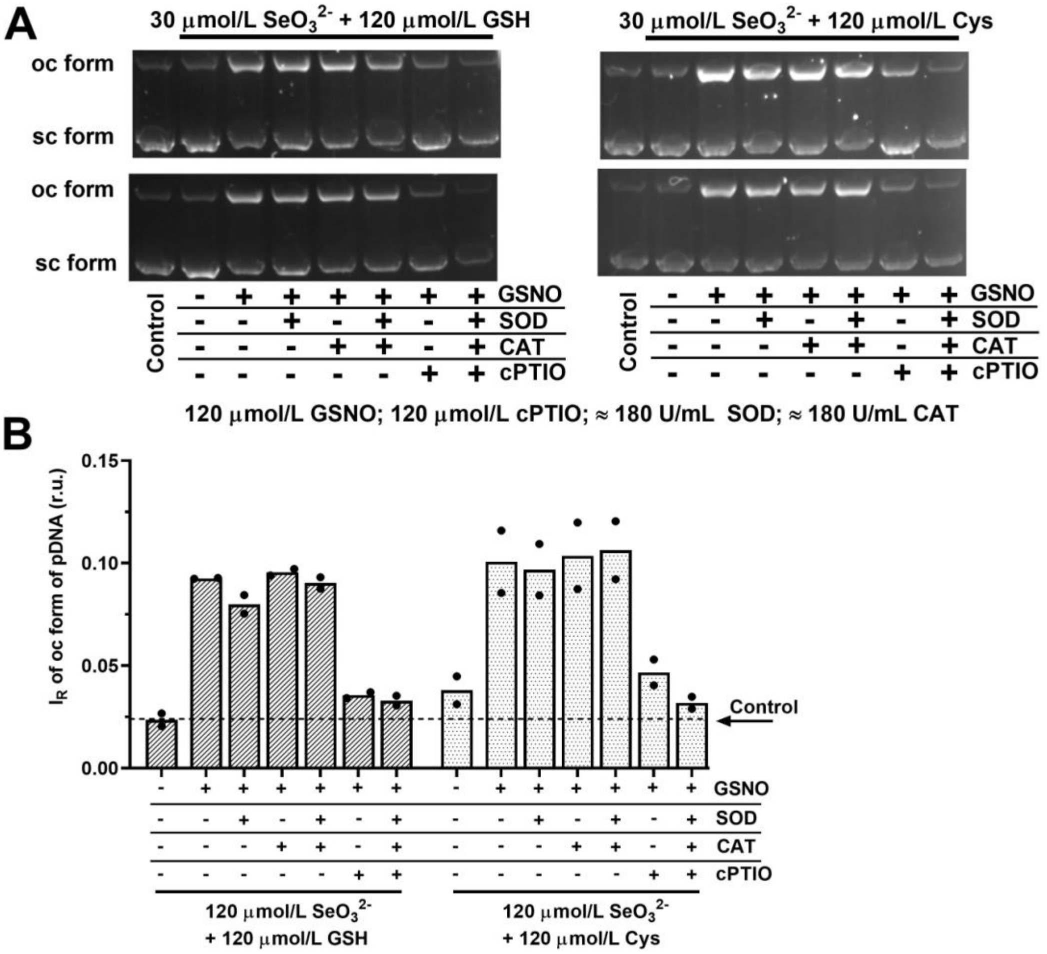

Fig. S14 Involvement of O_2^{-}, H_2O_2 and NO in pDNA cleavage induced by thiol/SeO_3^{2-}/GSNO. A. Agarose gel electrophoresis pDNA showing cleavage effects of mixtures of GSH (120 μmol/L; left panel) or Cys (120 μmol/L; right panel) with GSNO (120 μmol/L) and SeO_3^{2-} (30 μmol/L) in the presence of SOD (~180 U/mL), CAT (~ 180 U/mL) or the specific NO scavenger cPTIO (120 μmol/L). The lower and upper bands correspond to the supercoiled (sc) and open circle (oc) forms of pDNA, respectively. B. Quantification of pDNA cleavage, expressed as the proportion of the oc form. The presence of SOD or/and CAT had virtually no effect on the cleavage induced by thiol/SeO_3^{2-}/GSNO mixture, whereas cPTIO abolished the cleavage activity. The dotted line represents control samples without treatment. Data were obtained from pDNA gels (n = 2). The final concentration of pDNA was 0.2 μg in a 20 μL reaction mixture containing 25 mmol/L sodium phosphate buffer and 50 μmol/L DTPA, incubated at 37°C
